# Cadmium and Lead Exposure, Nephrotoxicity, and Mortality

**DOI:** 10.3390/toxics8040086

**Published:** 2020-10-13

**Authors:** Soisungwan Satarug, Glenda C. Gobe, David A. Vesey, Kenneth R. Phelps

**Affiliations:** 1 Kidney Disease Research Collaborative, The University of Queensland Faculty of Medicine and Translational Research Institute, Woolloongabba, Brisbane 4102, Australia; g.gobe@uq.edu.au (G.C.G.); David.Vesey@health.qld.gov.au (D.A.V.); 2 School of Biomedical Sciences, The University of Queensland, Brisbane 4072, Australia; 3 NHMRC Centre of Research Excellence for CKD.QLD, UQ Faculty of Medicine, Royal Brisbane and Women’s Hospital, Brisbane 4029, Australia; 4 Department of Nephrology, Princess Alexandra Hospital, Brisbane 4075, Australia; 5 Stratton Veteran Affairs Medical Center and Albany Medical College, Albany, NY 12208, USA; Kenneth.Phelps@va.gov

**Keywords:** cadmium, chronic kidney disease, lead, mortality, nephrotoxicity, threshold limit, tolerable intake level

## Abstract

The present review aims to provide an update on health risks associated with the low-to-moderate levels of environmental cadmium (Cd) and lead (Pb) to which most populations are exposed. Epidemiological studies examining the adverse effects of coexposure to Cd and Pb have shown that Pb may enhance the nephrotoxicity of Cd and vice versa. Herein, the existing tolerable intake levels of Cd and Pb are discussed together with the conventional urinary Cd threshold limit of 5.24 μg/g creatinine. Dietary sources of Cd and Pb and the intake levels reported for average consumers in the U.S., Spain, Korea, Germany and China are summarized. The utility of urine, whole blood, plasma/serum, and erythrocytes to quantify exposure levels of Cd and Pb are discussed. Epidemiological studies that linked one of these measurements to risks of chronic kidney disease (CKD) and mortality from common ailments are reviewed. A Cd intake level of 23.2 μg/day, which is less than half the safe intake stated by the guidelines, may increase the risk of CKD by 73%, and urinary Cd levels one-tenth of the threshold limit, defined by excessive ß_2_-microglobulin excretion, were associated with increased risk of CKD, mortality from heart disease, cancer of any site and Alzheimer’s disease. These findings indicate that the current tolerable intake of Cd and the conventional urinary Cd threshold limit do not provide adequate health protection. Any excessive Cd excretion is probably indicative of tubular injury. In light of the evolving realization of the interaction between Cd and Pb, actions to minimize environmental exposure to these toxic metals are imperative.

## 1. Introduction

Cadmium (Cd) and lead (Pb) are metals that have no biologic role in humans [1,2,3,4]. All of their perceptible effects are toxic [1,2,3,4]. Indeed, Cd and Pb are two of ten chemicals listed by the World Health Organization (WHO) as environmental pollutants of major public health concern [5]. Tissues and organs accumulate Cd and Pb because no excretory mechanism has evolved to eliminate these metals [6,7,8]. Consequently, tissue levels of Cd and Pb increase with age, as do risks of common ailments that are often viewed as outcomes of aging. Although the highest concentrations of Cd and Pb are found, respectively, in kidneys and bone, toxic effects of these metals are not confined to diseases of the kidney and skeleton [1,2,3,4,9,10]. It has been estimated that dietary intake of Cd, Pb, inorganic arsenic, and methylmercury have resulted in 56,000 deaths and more than 9 million disability-adjusted life-years worldwide [11]. For the nonsmoking population of adults, diet is the main exposure source of Cd and Pb [2,12,13,14,15,16].

Oxidative stress and inflammation have been identified as common toxic mechanisms of Cd and Pb even though neither metal undergoes a change in valence (redox inert) [17,18,19,20,21,22]. Both are primarily divalent [22,23,24]. In addition, Cd has a similar ionic radius to that of calcium (Ca) and electronegativity similar to that of zinc (Zn), and both Cd and Pb exhibit higher affinity than Zn for sulphur-containing ligands (Cd > Pb > Zn) [23,24,25,26]. Consequently, displacement of Zn and Ca and disruption of Zn and Cu homeostasis are other plausible toxic mechanisms [27,28,29,30,31,32,33,34,35]. All sulphur-containing amino acids, peptides and proteins with functional thiol (-SH) groups are potential ligands (molecular targets) for Cd and Pb. Examples include glutathione (GSH), numerous enzymes, zinc-finger transcription factors, and the metal-binding protein metallothionein (MT) [23,24,36]. Through Zn displacement, Pb impairs the activity of delta-aminolevulinic acid dehydratase (δ-ALAD), an enzyme required for the biosynthesis of heme, which is the functional group of hemoglobin, nitric oxide synthase, and cytochromes of the mitochondrial respiratory chain and xenobiotic metabolism [37]. Inhibition of the calcium-permeable acid-sensing ion channel may be the mechanism that accounts for the neurotoxicity of Pb [38,39].

The goal of this review is to provide an update on health risks associated with the low-to-moderate levels of environmental Cd and Pb to which most populations are exposed. High-dose exposure, which is relatively rare, is outside the scope of this review. We sought to establish dose–response relationships between ingested amounts of these toxic metals and parameters of tubular cell injury that have been associated with loss of glomerular filtration. This information is relevant to public health policy regarding advisable exposure limits. We discuss the interim safe intake level for Pb, the current tolerable dietary intake level for Cd, and the concept of the threshold level of urinary Cd. We describe the main dietary sources of Cd and Pb and the estimated intake levels of these metals among average and high consumers of tainted food. We highlight the utility of blood and urinary levels of Cd and Pb as indicators of internal accumulation of the metals. A connection is elaborated between Cd-induced tubulopathy and a decrease in glomerular filtration rate (GFR) to levels commensurate with chronic kidney disease (CKD). Epidemiologic data linking Cd and Pb exposure to enhanced CKD risk are summarized, as are data from longitudinal studies showing that Cd and Pb exposure may increase mortality from cancer and cardiovascular disease.

## 2. Health Risk Assessment of Chronic Exposure to Cadmium and Lead

### 2.1. The Critical Target of Toxicity

Long-term chronic exposure to Cd and Pb has been associated with distinct pathologies in nearly every tissue and organ throughout the body [1,2,3,4,14,25]. However, in health risk assessment, the kidney was considered to be the critical target of Cd toxicity [1,8], while the brain was the critical target of Pb toxicity [3,4,25]. Accordingly, dietary intake estimates associated with a significant increase in the risk of nephrotoxicity of Cd or neurotoxicity of Pb were used to derive a tolerable intake level. One method to evaluate whether a given food contaminant poses a health risk is to compare dietary intake estimated by total diet studies with the provisional tolerable weekly intake (PTWI), as established by the Joint Expert Committee on Food Additives and Contaminants (JECFA) of the Food and Agriculture Organization (FAO) and the WHO of the United Nations (FAO/WHO).

### 2.2. Tolerable Intake Levels

The PTWI for a chemical was defined as an estimate of the amount of a given chemical that can be ingested weekly over a lifetime without an appreciable health risk. The PTWI figures were first provided for Cd and Pb in 1989 and then amended in 1993 and 2010 [40,41]. The 1993 PTWI figures for Cd and Pb were 7 and 25 µg per kg body weight per week, respectively. In 2010, the PTWI for Cd was amended to a tolerable monthly intake (TMI) level of 25 μg per kg body weight per month. This intake level is equivalent to 0.83 μg per kg body weight per day or 58 μg per day for a 70-kg person [41]. The model for deriving PTWI and TMI of Cd was based on elevated β_2_-microglobulin (β_2_MG) excretion as the sole evidence of nephrotoxicity [41]. In Section 3.1, we provide current Cd intake levels in various countries and their sources.

For Pb, the previously established PTWI of 25 μg per kg body weight per week was withdrawn because it did not afford health protection [41]. A new tolerable Pb intake level could not be established as dose–response analyses indicated that no threshold levels exist for neurotoxicity of Pb. Thus, no amount of Pb intake is safe, and no tolerable Pb intake level has been officially identified. However, the U.S. Food and Drug Administration (FDA) has proposed a dietary Pb intake level of 12.5 μg/day as an interim safe intake level for the general population of adults [42,43]. This intake level corresponds to a blood concentration of Pb ([Pb]_b_) of 0.5 μg/dL, which has not been found to be associated with an adverse effect in adults in any epidemiologic studies. In Section 3.1, we provide current Pb intake levels in various countries and their sources.

### 2.3. Urinary Cd Threshold Level

A urinary Cd excretion rate (E_Cd_) of 5.24 μg/g creatinine was adopted as a threshold limit [41]. However, the established threshold level is questionable. Chronic environmental exposure to low-level Cd, producing urinary Cd one-tenth of the conventional threshold, has been associated with deterioration of kidney function, as assessed with estimated GFR (eGFR) [44,45,46]. A urinary Cd concentration ([Cd]_u_) as low as 1 μg/L, corresponding to blood Cd concentration ([Cd]_b_) of 0.5 μg/L, was associated with an increased risk of eGFR less than 60 mL/min/1.73 m^2^ [44,47]. It can be argued that risk of nephrotoxicity of any toxicants, Cd and Pb included, should be based on eGFR, which is a reliable measure of kidney function and diagnosis and staging of CKD [48,49,50]. A dose–response analysis of urinary Cd and eGFR, rather than of urinary Cd and β_2_MG, indicates that Cd-induced nephrotoxicity occurs at a much lower E_Cd_ than previously thought [12,51,52,53,54]. We believe that the established TMI for Cd is not protective of kidneys, just as the 1993 PTWI for Pb does not prevent neurotoxicity. The 1993 PTWI for Pb has now been withdrawn [41]. In Section 4.3.4. we provide an in-depth analysis of β_2_MG excretion in Cd nephropathy.

## 3. Exposure Sources and Dietary Intake Estimates

For the general nonsmoking population of adults, the diet is the major exposure source of both Cd and Pb. In this section, both natural and anthropogenic sources of Cd and Pb in the human diet are highlighted. In addition, a reliable dietary assessment and food safety monitoring method, such as a total diet study, is discussed, and estimated intake levels of Cd and Pb derived from recent total diet studies in various countries are provided.

### 3.1. Environmental Sources of Cadmium and Lead

Volcanic emissions, fossil fuel and biomass combustion, and cigarette smoke are sources of Cd and Pb released as CdO and PbO [55,56,57,58,59]. Experimental studies have shown that inhaled CdO and PbO are more bioavailable than oral Cd and Pb [60,61,62,63]. Typically, potable water is not a source of Cd or Pb, except in cases where significant amounts of Pb plumbing have been used, as occurred in the recent Flint, Michigan, water crisis [64,65].

Years of production and industrial use of Cd and Pb have mobilized these metals from nonbioavailable geologic matrices to biologically accessible sources from which they can enter food chains [55]. Like all other metals, Cd and Pb are not biodegradable and thus can persist indefinitely in the environment [55]. The use of contaminated phosphate fertilizers has also added these toxic metals to agricultural soils [6,7], causing a further increase in Cd and Pb in the food chain [66,67,68]. Livestock that graze on contaminated pastures can accumulate Cd in the kidney and liver at levels that make these organs unsafe for human consumption [69]. In Pb-exposed cattle, blood Pb levels correlated with levels of Pb in liver, bone and kidney, but not in brain or skeletal muscle (beef) [70]. Of note, a detectable amount of Pb was found in beef at a blood Pb concentration of 4.57 μg/dL. This blood Pb level was close to the exposure limit for neurotoxicity of Pb in children (5 µg/dL) [71]. Molluscs and crustaceans accumulate Cd and are also notorious hyperaccumulators of other metals [72,73,74,75]. For most species, fish muscle does not appear to be a significant source of Cd and Pb, but there are exceptions, as indicated in Section 3.1 [76].

In a similar manner to molluscs and crustaceans, plants have the propensity to concentrate Cd and Pb from the soil. Plants have evolved multiple metal detoxification mechanisms, including an array of metal-binding ligands such as MT, phytochelatins (PCs), other low-molecular-weight thiols, GSH, cysteine, γ-glutamylcysteine, and cysteinylglycine [77,78,79]. As Cd exerts toxicity in the “free” ion or unbound state, complexes of Cd and metal-binding ligands, such as CdMT and CdPC, are viewed as detoxified forms [80]. Accordingly, the various types of metal-binding ligands render plants capable of tolerating levels of Cd and Pb that are toxic to animals and humans.

Owing to their phylogenic characteristics, tobacco, rice, other cereal grains, potatoes, salad vegetables, spinach, and Romaine lettuce accumulate Cd more efficiently than other plants [81]. An outbreak of “itai-itai” disease, a severe form of Cd poisoning from contaminated rice, serves as a reminder of the health threat from Cd contamination of a staple food crop [82].

### 3.2. Total Diet Studies and Dietary Intake Estimates

Reliable methodology is vitally important to assess the levels of contaminants in commonly eaten foods and to set food safety standards. The total diet study has been widely used by authorities to estimate intake levels and identify sources of Cd and Pb in the human diet [83,84,85,86,87]. It is also known as the “market basket survey” because samples of foodstuffs are collected from supermarkets and retail stores to determine levels of nutrients, food additives, pesticide residues and contaminants [2,83,84,85,86,87]. It serves as a food safety monitoring program that provides a basis to define a maximally permissible concentration of a given contaminant in a specific food group.

In a typical total diet study, an intake level of a given contaminant from a study food item (rice as an example) is computed based on an amount of the food item consumed per day and the concentration of a contaminant in the rice samples that are analyzed in a study. The median and 90th percentile concentration levels of a contaminant are used to represent the intake levels of a contaminant by average and high consumers, respectively [88].

Table 1 summarizes most recent total diet studies showing intake levels of Cd among adult consumers in China [89,90,91], Korea [92,93], Germany [94], Spain [95,96] and the U.S. [97,98,99] along with the list of foods that contributed significantly to total intake of the metal. Table 1 summarizes also food products that contributed significantly to total intake of Pb and the estimated intake levels of the metal among adult consumers in China [89,90,91], Korea [92], Germany [100], Spain [95] and the U.S. [84]. Furthermore, Cd intake levels estimated for consumers in Sweden [88] France [101], Belgium [102] and a region with Cd pollution of Japan [103] are provided.

#### 3.2.1. Estimated Cadmium Intake Levels in Various Populations

In a recent total diet study in China, the Cd intake among average consumers was 32.7 μg/day, with rice and vegetables being the main sources [89]. In Mongolia, potatoes were the main source of Cd, contributing 24% of the total Cd intake [89]. Nori, peanuts, squid, cuttlefish and mushrooms had relatively high Cd contents [90,91].

The Cd intake level among average consumers in South Korea was 12.6 μg/day [92]. Cereals and vegetables, beverages, fruits and nuts, and dairy products (milk included) were the main dietary sources. Cereals, oily seeds and fruits, and vegetables had relatively high Cd concentrations. A higher average Cd intake of 22 μg/day was reported in another Korean study (*n* = 1245), where Cd intake in gastric cancer cases was compared to noncancer controls [93]. An average amount of rice consumed by the control group was 587.3 g/day versus 610.9 g/day in gastric cancer cases. Rice was the major contributor (40.3%) to total Cd intake, followed by squid (11.8%), eel (11.0%), crab (8.6%), shellfish (3.6%), kimchi (Korean cabbage; 3.5%) and seaweed (3.5%).

The Cd intake levels among average and high consumers in Germany were 14.6 and 23.5 μg/day, respectively [94]. Cereals and vegetables were the main Cd sources, followed by beverages, fruits and nuts, and dairy products (milk included). Cereals, oily seeds and fruits, and vegetables had relatively high Cd contents [94].

The Cd intake among average consumers in Spain was 7.7 μg/day, with cereals and fish as the main sources, contributing to 38% and 29% of total Cd intake, respectively [95]. In another dietary study of 281 postmenopausal women in Spain, an average Cd intake was 30 (range, 20−41) μg/day [96]. These data illustrate that when the total diet study methodology is used, Cd intake from the diet showed a little variation and, in many cases, probably represented an underestimation of actual Cd intake.

The Cd intake among average consumers in the U.S. was 4.63 μg/day. Cereals and bread, leafy vegetables, potatoes, legumes and nuts, stem/root vegetables, and fruits contributed, respectively, to 34%, 20%, 11%, 7% and 6% of total intake [97]. Spaghetti, bread, potatoes and potato chips were the top three Cd sources, followed by lettuce, spinach, tomatoes, and beer. Lettuce was an important Cd source for whites and blacks. Tortillas and rice were the main Cd sources for Hispanic Americans, Asians and some other ethnicities. However, a higher dietary Cd intake of 10.9 μg/day was recorded in the U.S. Women’s Health Initiative study [98]. The average Cd intake of 4.63 μg/day by adults in the U.S. was close to a median Cd intake of 5 μg/day reported for consumers in Northern Italy, where cereals, vegetables and sweets were the main Cd sources [99].

It is noteworthy that the average Cd intake in Sweden (10.6 μg/day), France (11.2 μg/day) and Belgium (9.8 μg/day) was higher in all of these countries than the average Cd intake in the U.S. of 4.63 μg/day [88,101,102]. For average consumers in France, bread and potato-based products contributed, respectively, to 35% and 26% of total Cd intake, while potatoes and wheat combined contributed to 40−50% of Cd intake among average consumers in Sweden. Likewise, cereal products and potatoes were the main sources, contributing more than 60% to total Cd intake among average consumers in Belgium [102]. For high consumers in Sweden, average Cd intake was 23 μg/day, with seafood (shellfish) and spinach being additional Cd sources [88]. For high consumers in France, Cd intake was 18.9 μg/day, with additional Cd coming from molluscs and crustaceans [101]. Cd contents in molluscs and crustaceans, offal, sweet and savoury biscuits and cereal bars, and chocolate in a French total diet study were 0.167, 0.053, 0.030, and 0.029 mg/kg, respectively.

In summary, the average Cd intake levels in China (32.7 μg/day), Korea (12.6 μg/day), Germany (14.6 μg/day), Spain (7.7 μg per day) and Sweden, France and Belgium (range, 9.81−11.2 μg/day) were all higher than the average Cd intake in the U.S (4.63 μg/day) [88,101,102]. For average consumers, staple foods were the main Cd sources. Seafood (shellfish), offal, spinach, lettuce and chocolate were additional sources of Cd among high consumers. These estimated Cd intake levels did not exceed a current FAO/WHO tolerable intake level of 58 μg/day [41]. However, evidence for adverse health effects has emerged from cross-sectional and longitudinal studies of populations from these countries, in which urinary and/or blood Cd levels were used to quantitate exposure levels (Section 5). Thus, the utility of dietary intake estimates in health risk assessment is questionable. In another example, the total diet study undertaken in two areas of Japan affected by Cd pollution reported the median Cd intake levels in female farmers residing in the two areas as 47.8 and 55.7 µg/day [103]. These estimated Cd intake levels in Cd-polluted areas were lower than the FAO/WHO tolerable intake level, but adverse health effects were observed [103].

#### 3.2.2. Estimated Lead Intake Levels in Various Populations

The Pb intake level among average consumers in China was 35.1 μg/day, with cereals, meats, vegetables, beverages and water as the main sources [89]. These food and beverage items together contributed to 73.26% of total Pb intake [89]. Kelp, nori, processed and preserved soybean, meat, and fungus products had relatively high Pb concentrations [90]. For the population of Jiangsu province, a higher mean Pb intake level of 73.9 μg/day was reported, with cereals and vegetables as the main sources, contributing to 57% of total Pb intake [91].

The Pb intake level among average consumers in South Korea was 9.8 μg/day [92]. High levels of Pb were found in a range of products, notably seaweed, shellfish and crustaceans, molluscs, fish, sugar and sugar products, and beverages (fruit juice, carbonated fruit juice, carbonated drinks, sports drinks and coffee).

The Pb intake levels among average and high consumers in Germany were 37.1 and 50.4 μg/day, respectively [100]. Beverages, vegetables, fruits and nuts and cereals were the main Pb sources. Foods with relatively high Pb concentrations were meat (offal included), fish (seafood), vegetables and cereals [100].

The Pb intake level among average consumers in Spain was 14.7 μg/day, with cereals as the main source at nearly half (49%) of total intake [95]. Sweeteners and condiments, vegetable oils, meat, and fish had relatively high Pb concentrations [95].

The Pb intake levels among average and high consumers in the U.S. were 1.7−5.3 and 3.2−7.8 μg/day, respectively [84]. Grains, beverages, vegetables, dairy products, fruits, meat, and poultry plus fish contributed to 24.1%, 14.3%, 10.7%, 9.7%, 9.3% and 3.4% of total intake, respectively. Foods with relatively high Pb concentrations were chocolate syrup, liver, canned sweet potatoes, brownies, low-calorie buttermilk, salad dressing, raisins, English muffins, canned apricots, milk chocolate, candy bars, chocolate cake, chocolate chip cookies, wine and oat ring cereal.

In summary, average Pb intake in China (35.1 μg/day) was close to the level of intake in Germany (37.1 μg/day). These Pb intake levels were higher than the intake figures estimated for average consumers in Spain (14.7 μg/day), Korea (9.8 μg/day) and the U.S. (1.7−5.3 μg/day). Pb intake from the diet was highest (50.4 μg/day) among high consumers in Germany. To date, a tolerable Pb intake level has not been identified after a tolerable intake level of 25 µg per kg body weight per week was withdrawn in 2010 [41]. An interim tolerable intake level for Pb of 12.5 μg per day has been proposed for the population of adults by the U.S. FDA [42,43]. Based on this interim safe-intake figure (12.5 μg/day), dietary Pb intake levels among average consumers in China, Germany and Spain could be considered as excessive and may pose a significant health risk.

### 3.3. Absorption of Cadmium and Lead: An Overview

As the body does not synthesize nor break down metals, transporter systems and pathways have evolved to acquire all required elements, notably calcium (Ca), zinc (Zn), manganese (Mn), copper (Cu), and iron (Fe), from exogenous sources [104]. These metal transporters and pathways also serve as routes of entry for toxic metals Cd and Pb in the diet [105,106,107,108,109,110]. Early work suggested that the iron (Fe^2+^) transporter, divalent metal transporter1 (DMT1), was a likely route of entry for Cd and Pb [111,112]. Later, it was demonstrated that Pb entered enterocytes through a mechanism that was independent of DMT1 [113,114]. Additionally, although DMT1 has the same high affinity for Cd^2+^ as it does for Fe^2+^ [112], ferroportin 1 (FPN1) exports iron but not Cd [115]. Calbindin-D28k, a calcium-binding protein, may transport Cd to the basolateral membrane of enterocytes and may export Cd into the portal blood circulation [110,116].

Receptor-mediated endocytosis and transcytosis are the likely mechanisms for absorption of the Cd–metallothionein complex (CdMT) and the Cd–phytochelatin complex (CdPC) [117,118,119]. The specific metal transporters, carriers and receptors that have been implicated in the absorption of Cd and/or Pb include DMT1, a Zrt- and Irt-related protein (ZIP) of the zinc transporter family, the Ca^2+^-selective channel TRPV6, and the human neutrophil gelatinase-associated lipocalin (hNGAL) receptor [110,120]. ZIP14 and TRPV6 are highly expressed by intestinal enterocytes [107,108,109,120].

### 3.4. The Kinetics of Cadmium and Lead in the Human Body

**Figure 1:**
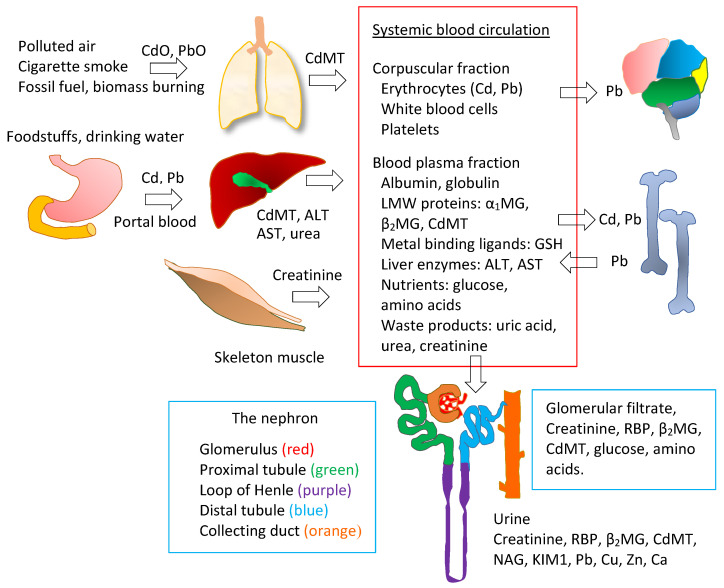
Entry routes, distribution, storage and urinary excretion of cadmium and lead.

Figure 1 outlines exposure sources of Cd and Pb, their entry routes, tissue distribution, storage organs and targets of toxicity. 

Absorbed Cd and Pb are transported to the liver. Inhaled CdO and PbO are transported to the lungs. Cd induces the synthesis of MT in both liver and lung, and CdMT is formed. Hepatic and pulmonary CdMT are later released and contribute to [CdMT]_b_. Pb does not induce MT in the liver or lung, and it binds presumably to GSH and other thiols in these organs. The fractions of absorbed Cd and Pb that are not taken up by the liver in the first pass then reach systemic circulation and are taken up by tissues and organs throughout the body, kidneys included. Pb is taken up mostly by bone and is later released, contributing to [Pb]_b_. CdMT from all sources (liver, lung, intestine) passes glomerular membrane filtration, and the filtered CdMT is reabsorbed by proximal tubular cells. Small fractions of Cd and Pb in the body are excreted in urine. [Cd]_u_ provides a useful indication of kidney injury and kidney burden. [Pb]_u_ serves as a proxy for [Pb]_p_.

Oxides of Cd and Pb in the air reach the lung, prompting an increase in the synthesis of MT, and CdMT is formed in situ [121]. Presumably, the induction of MT synthesis in the lung is attributable to Cd only because Pb is a weak inducer [122]. The pulmonary CdMT is later released into systemic circulation and redistributed to the kidneys [123,124]. Of note, while inhaled Cd increased pulmonary MT synthesis, a study in mice has shown that low-dose oral Cd did not induce pulmonary MT [125].

Cd and Pb in the diet are transported via the hepatic portal system to the liver, where Cd induces copiously the synthesis of MT, to which it becomes tightly bound as CdMT [126]. Pb does not induce MT synthesis in the liver. An increase in hepatic MT protein levels was a result of elevated plasma levels of interleukin 6 that followed Pb administration [127]. Of note, hepatic MT synthesis is intensified in rats given Pb plus Cd, although oral Pb alone does not affect the synthesis of MT in the liver [128]. Presumably, in hepatocytes, Pb initially forms complexes with GSH [24]. A small fraction of hepatic Cd and Pb is excreted in faeces via bile or may be reabsorbed and returned to the liver [129]. Hepatic CdMT is also continuously released into systemic circulation. This hepatic process contributes to [Cd]_b_ and the redistribution of Cd (as CdMT) to kidneys long after exposure cessation. In this manner, the liver serves as a reservoir of Cd. The fraction of dietary Cd and Pb not taken up by hepatocytes in the first pass reaches systemic circulation and is taken up by tissues and organs throughout the body. Pb is preferentially taken up by bone, where it is stored and later released [130]. Thus, bone serves as a reservoir of Pb, and this organ contributes to [Pb]_b_, as is discussed further in Section 4.2.

Through systemic circulation, CdMT and CdPC of dietary origin and intestinal and hepatic CdMT [126] all reach the kidneys, where they are filtered by glomeruli by virtue of their small molecular weights [8]. Once they pass the glomerular membrane into the tubular lumen, they are then subsequently reabsorbed by proximal tubular epithelial cells as these cells are equipped with protein internalization mechanisms [131,132,133,134]. Due to a lack of biologically active mechanisms to eliminate excess metals, nearly all reabsorbed Cd is retained in the kidneys.

**Figure 2:**
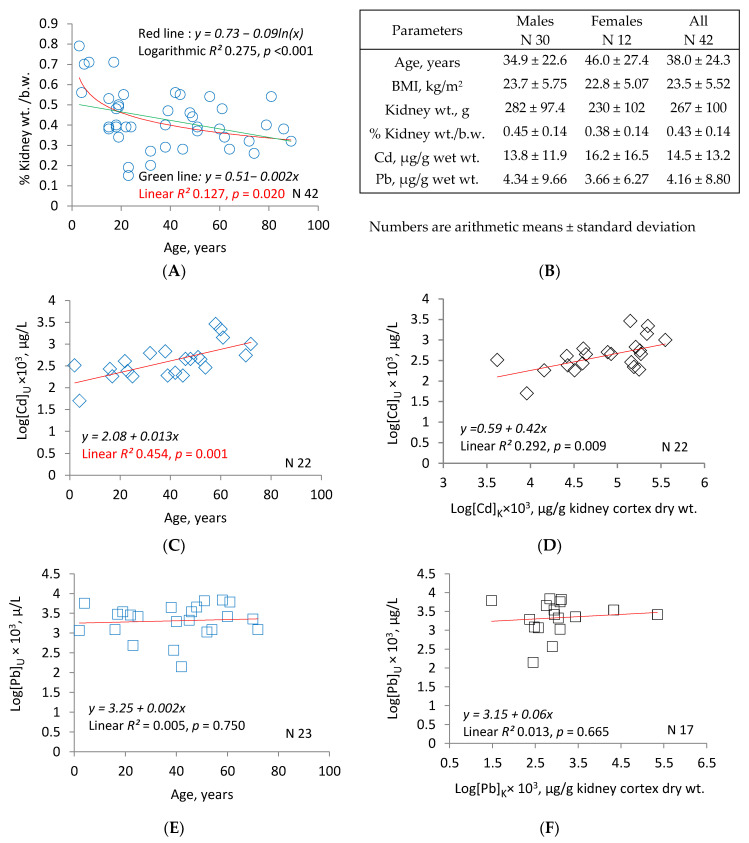
Cadmium and lead accumulation levels in kidneys and their levels in urine.

In summary, ingested Cd is mostly reabsorbed and sequestered in kidneys [126,131,132,133,134], while ingested Pb is taken up and retained in bone [135]. Only a small fraction of Cd and Pb in the kidneys is excreted in urine (Section 3.4, Figure 2). An in-depth discussion on the source of excreted Cd is provided in Section 4.3.2. In effect, the kidney Cd content reflects cumulative lifetime exposure, while bone Pb is an indicator of Pb body burden.

### 3.5. Cadmium and Lead Accumulation in Kidneys and Urinary Excretion: Australian Experience

Figure 2 provides data on Cd and Pb levels in kidney cortex samples ([Cd]_k_, [Pb]_k_) from a subgroup of an Australian autopsy study (*n* = 42) [136,137]. The mean age, body mass index (BMI) and kidney weight were 38 years, 23.5 kg/m^2^ and 267 g, respectively. Kidney weight relative to body weight showed a decrease in old age (Figure 2A). The means of [Cd]_k_ and [Pb]_k_ were 14.5 and 4.16 μg/g kidney cortex wet weight, respectively. Thus, the kidney cortex accumulated 3.5 times greater amounts of Cd than Pb on a weight basis and 6.4 times higher amounts of Cd than Pb on a molar basis (Figure 2B). In an analysis of Cd and Pb concentrations in urine samples ([Cd]_u_, [Pb]_u_) from 17–23 urinary bladders of subjects in this subgroup, [Cd]_u_ increased linearly with age and [Cd]_k_ (Figure 2C,D). Distinctly, [Pb]_u_ did not correlate with age or [Pb]_k_. The correlation between [Cd]_u_, age and [Cd]_k_ supports the use of [Cd]_u_ as a kidney burden indicator. The mean [Pb]_k_ of 4.16 μg/g wet weight observed in this Australian study was much higher than the median [Pb]_k_ of 0.08 μg/g wet weight found in Swedish kidney transplant donors (*n* = 109, 24–70 years, median 51), but there was only a small difference in [Cd]_k_ in Australian and Swedish studies (14.5 vs. 12.9 μg/g wet weight) [138].

Figure 2A is a scatterplot that relates % kidney weight to body weight ratios to age in a subgroup of an Australian autopsy study (*n* = 42, mean age 38 years). Figure 2B provides data on age, BMI, kidney weight and Cd and Pb contents in kidney cortex ([Cd]_k_, [Pb]_k_). Figure 2C,D are scatterplots showing significant correlations between [Cd]_u_ versus age and [Cd]_u_ versus [Cd]_k_, respectively. These correlations support the utility of [Cd]_u_ in kidney burden assessment. Miniscule correlations between [Pb]_u_ versus age and [Pb]_u_ versus [Pb]_k_ are indicated by scatter plots in Figure 2E,F, respectively.

## 4. Dosimetry and Nephrotoxicity Assessment

The dogma in health risk assessment states that for a given toxicant to pose a health risk, it must enter the body to reach its critical target of toxicity. The levels of a toxicant in body fluid and tissues and organs may serve as dosimetric matrices. In this section, we discuss the utility of urine, whole blood, blood plasma (serum), and erythrocytes in the assessment of Cd and Pb body burden. The manifestation of nephrotoxicities such as the leakage of intracellular proteins into the filtrate, a reduction in glomerular filtration rate, and impaired reabsorption of filtered proteins is discussed.

### 4.1. Cadmium Dosimetry

#### 4.1.1. Blood Cadmium Versus Urinary Cadmium

In the studies examining decreases in [Cd]_b_ and [Cd]_u_ in groups of Cd-exposed Swedish workers after exposure had ceased, the estimated half-life values varied from 75 to 128 days for the fast component, representing [Cd]_b_ [139,140]. For the slow component, representing Cd in tissues, the estimated half-life values varied from 7.4 to 16.0 years. A comparable half-life figure of 14 years (range, 9−28 years) was derived from a Japanese study [141]. In a separate analysis, the half-life of whole-body Cd in Japanese men with lower exposures ([Cd]_u_ < 5 µg/L) was 23.4 years, nearly two-fold longer than the half-life figure of 12.4 years, estimated for those with higher exposures [142]. A whole-body half-life of Cd over 45 years was estimated based on data from Swedish kidney transplant donors, who had much lower [Cd]_u_, [Cd]_b_ and [Cd]_k_ than those in Japanese studies [143]. Collectively, these findings indicate that Cd is mostly retained in the body, particularly the kidneys, in low-level exposure situations, while there is a substantial loss of [Cd]_k_ due to Cd-induced tubular injury in chronic high-exposure conditions [54].

The half-life of whole-body Cd falls in the same range as estimated half-life for Pb in bone (Section 4.2). Apparently, however, the half-life of blood Cd (between 75 and 128 days) is much longer than the 10-day half-life of blood Pb [144]. The estimated long half-life of [Cd]_b_ could be due to continuing contribution to [Cd]_b_ from tissues and organs, notably, the liver and lungs. To derive a half-life figure specific to exogenously derived Cd requires knowledge of contribution from the main endogenous sources, liver, lung and bone included, as depicted in Figure 1. Thus, at any given time, [Cd]_b_ is considered to reflect recent exposure together with a continuing contribution from whole-body Cd, especially the liver and lungs.

Distinct from [Cd]_b_, urinary excretion of Cd (E_Cd_) is an indicator of cumulative lifelong exposure. E_Cd_ varies directly with [Cd]_k_ (see Figure 2) and the determinants of [Cd]_k_, such as age, gender, smoking, metalwork history and level of regional Cd pollution [8,136,145,146,147,148]. In a Swedish study, the highest Cd concentrations in copper smelter workers and the controls were seen in kidneys, followed by liver, lung and brain [148]. Of note, E_Cd_ showed stronger and more consistent associations with nephrotoxicity than [Cd]_b_. E_Cd_ is discussed further in Section 4.3.2.

In an autopsy study in Poland, E_Cd_ of 1.7 µg/g creatinine corresponded to [Cd]_k_ of 50 µg/g [145]. In another Swedish study, kidney transplant donors (*n* = 109) had a mean [Cd]_k_ of 12.9 μg/g, and the “urine-to-kidney Cd ratio” of 1:60. Accordingly, E_Cd_ of 0.42 μg/g creatinine corresponded to [Cd]_k_ of 25 μg/g wet kidney weight [146]. In another study of kidney transplant donors, the means for E_Cd_, [Cd]_b_, and [Cd]_k_ in women were 0.34 μg/g creatinine, 0.54 μg/L and 17.1 μg/g kidney wet weight, respectively [147]. The corresponding figures in men were 0.23 μg/g creatinine, 0.46 μg/L and 12.5 μg/g, all of which were lower than in women [147].

#### 4.1.2. Cadmium in Erythrocytes Versus Blood Plasma (Serum)

The vast majority of [Cd]_b_ appears to be concentrated in the cytosol of red blood cells. The chloride/bicarbonate anion exchanger ([Cl−/HCO3−], AE1, SLC4A1), is the major Cd transporter responsible for its uptake [149,150,151]. High- and low-affinity “iron” transport mechanisms have also been suggested to mediate Cd uptake [152]. Of interest, these transport mechanisms are also responsible for Pb and Zn uptake by erythrocytes [152,153,154]. Although Cd is concentrated in red blood cells, the toxicity of Cd in these cells has not been examined. One potential toxic effect is that Cd causes erythrocytes to undergo suicidal death, known as eryptosis, the mechanism by which such injured cells are removed [155,156]. Enhanced eryptosis, seen in smokers, has been linked to oxidative stress and inflammation [157].

The Cd that remains in the plasma is bound to the amino acid histidine and proteins such as MT, pre-albumin, albumin, α_2_-macroglobulin, and immunoglobulins G and A [126,158,159]. A range of low molecular weight thiols, including GSH, cysteine, cysteinylglycine, homocysteine, and γ-glutamylcysteine, are also possible carriers of Cd [160,161,162]. The total concentrations of these low molecular weight thiols are in the low µM range (12–20 µM), while albumin thiol is more abundant (~0.6 mM). As Cd in plasma is readily exchangeable with other metals in target tissues, [Cd]_p_ is more relevant than [Cd]_b_ (Cd in erythrocytes) to toxic injury in those tissues. This may explain the conflicting results when different indicators of exposure were used. For instance, an association was not observed between breast cancer risk and [Cd]_b_ [163], while [Cd]_u_ has been consistently associated with a significant increase in risk for cancer [164].

Presently, plasma/serum Cd is rarely used in health risk assessment. The true distribution of Cd in whole blood and serum (plasma) remains to be fully characterized. The relationship between [Cd]_b_ and plasma Cd at varying exposure levels is not known, nor is there a way to predict plasma Cd from whole blood/erythrocyte Cd data that are abundantly available. Nonetheless, the utility of the serum Cd concentration has been demonstrated in the studies linking Cd to an increased risk of respiratory disease in a representative U.S. population. Serum Cd levels ≥ 0.73 µg/L were associated with a 2.5-fold increase in the risk of obstructive lung disease among participants in NHANES 2007–2010 [165], while serum Cd levels in the highest quartile were associated with a 2.8-fold increase in the risk of recurrent wheeze and asthma in NHANES 2007–2012 participants aged 20–79 years [166].

### 4.2. Lead Dosimetry

#### 4.2.1. Blood Lead Versus Bone Lead

Blood Pb ([Pb]_b_) constitutes less than 2% of the total body content of Pb. Ninety-nine percent of blood Pb is found in the cytosol of erythrocytes, while 1% or less is present in plasma [167,168,169,170]. However, [Pb]_b_ is still the most frequently used marker of Pb exposure in epidemiologic studies because higher quantities are detectable by common analytical instruments compared with measurements of Pb in bone and urine. [Pb]_b_ of 5 μg/dL is recommended as an actionable level or an excessive exposure level in infants and children, while [Pb]_b_ of 20 μg/dL is adopted as an excessive exposure level in workplace settings [71].

At any given time, [Pb]_b_ reflects recent intake plus the contribution from bone Pb (cumulative lifetime exposure). In children, the bone contribution is more than 90%, while the bone contribution to [Pb]b in adults ranges from 45% to 55% [171,172,173]. An estimated half-life of [Pb]_b_ was 10 days (9.96 ± 3.92) in a study of Chinese children with Pb toxicity, in whom both bone Pb and [Pb]_b_ were measured [144]. This estimated half-life of [Pb]_b_ is much shorter than the previously estimated figures that ranged from 8–11 months to 2–3 years [174]. A half-life of as long as 2 years for [Pb]_b_ was suggested in a study of the time required for [Pb]_b_ to fall by 50% in Pb-exposed children undergoing chelation therapy [175]. The differences in [Pb]_b_ half-life figures are due mostly to a correction for bone contribution in a recent study [144].

Bone Pb constitutes approximately 94% and 74% of the total body burden of this metal in adults and children, respectively [144,176,177,178]. The half-life of bone Pb varies from 10 to 20 years [179,180,181,182]. In a study of Pb accumulation in different types of bone from Swedish copper–lead smelter workers and controls, the highest Pb concentration was found in finger bone, followed in order by vertebrae, iliac crest and sternum [183]. Among soft tissues, Pb concentration was highest in the liver, followed by kidney, lung and brain [184]. Consistent with this study, an Australian study reported that liver contained Pb levels two times higher than kidney cortex: the mean hepatic and kidney cortex Pb levels were 0.19 and 0.09 µg/g wet tissue weight, respectively [137]. Bone Pb levels correlated with serum Pb levels in adults who were exposed to Pb in the workplace [185].

In environmental exposure scenarios, Pb in tibia showed stronger and more consistent associations with neurotoxicity than blood Pb. This supports bone Pb to be a better biomarker of cumulative dosimetry or the body burden than blood Pb [186,187,188]. In addition to the tibia, Pb in the patella was associated with declines in cognitive test scores in older persons [189]. In a prospective cohort study of men, chronic, low-level Pb exposure, assessed with bone Pb, has been linked to hypertension [190] and incident coronary heart disease [14]. The Western dietary patterns have also been linked to elevated bone and blood Pb levels in men [13,14].

#### 4.2.2. Plasma (Serum) Lead Versus Urinary Lead

For the same reason as plasma Cd (see Section 4.1.2), [Pb]_p_ is more relevant than [Pb]_b_ to toxicity in tissues/organs. The superiority of plasma Pb has been demonstrated in an early study of Pb-exposed Japanese workers, in which the strength of correlations between [Pb]_p_ and urinary indicators of adverse effect (the inhibition of heme biosynthesis), namely, urinary coproporphyrin and urinary delta-aminolevulinic acid (ALA), was stronger than the correlations of [Pb]_b_ with these indicators [191]. Although only 1% of whole-blood Pb is in the plasma fraction, this percentage increases sharply in high-exposure conditions, a phenomenon that suggests the binding capacity of the erythrocytes has been exceeded. A linear relationship between [Pb]_p_ and [Pb]_b_ has been demonstrated using data from Pb-exposed workers [182,192]. Likewise, a linear relationship between [Pb]_p_ and [Pb]_b_ was observed in a study of U.S. women of child-bearing age [183].

Notably, however, the measurement of [Pb]_p_ can be problematic because hemolysis causes a spurious increase in [Pb]_p_. [Pb]_u_ correlated more closely with [Pb]_p_ than [Pb]_b_ in a study of Pb-exposed workers in Japan [191]. [Pb]_u_ may, thus, be used as a proxy for [Pb]_p_. The utility of urine in monitoring Pb exposure has been illustrated in studies in Japan [193], Belgium [194], China [195], Thailand [12] and the U.S. [196]. In a Chinese study, workers in a lead–zinc mine had higher [Pb]_u_, [Cd]_u_ and urinary 8-hydroxydeoxyguanosine, an indicator of oxidative stress, compared with those who worked in a steel smelting plant [195].

In a Thai study, the 75th percentile level of [Pb]_u_ was associated with a 2.3-fold increase in the risk of reduced eGFR [12]. This finding is reminiscent of studies relating E_Cd_ to eGFR (Section 4.3.2). In a follow-up study of 5316 participants in NHANES 1999–2010, [Pb]_u_ levels > 1.26 μg/L were associated, respectively, with 1.79- and 6.60-fold increases in mortality from all causes and cancer [196].

### 4.3. Assessment of Cadmium Nephrotoxicity

As Figure 1 indicates, Cd enters the body from contaminated food, tobacco smoke, and polluted air. It is transported through the gut and lungs to the bloodstream, where it is bound primarily to albumin and taken up by red blood cells ([126], see Section 3.4). Most circulating Cd is eventually transferred to hepatocytes, which then synthesize the protein MT and store Cd in complexes of CdMT. As hepatocytes die, these complexes are released to the circulation, filtered by glomeruli, and reabsorbed primarily or entirely by proximal tubular cells [197,198]. After lysosomes within these cells degrade the complexes, Cd enters the cytoplasm, where it again induces the synthesis of MT, to which it is subsequently bound. Cd that remains free is believed to induce the release of copper (Cu), a transition metal, from MT; Cu then promotes the formation of reactive oxygen species, which inflict cellular injury [31,199]. The intensity of this injury is thought to be related to the concentration of free Cd in the cytoplasm [200,201]. As no mechanism exists for discharging Cd from intact tubular cells, the metal continues to accumulate in those cells as long as hepatocytes release CdMT to the bloodstream [202,203].

Commonly studied clinical expressions of tubular Cd toxicity include leakage of intracellular proteins into the filtrate, excretion of Cd itself, the estimated glomerular filtration rate (eGFR), and impaired reabsorption of small filtered proteins. Extreme toxicity also compromises reabsorption of filtered substances that are cotransported with sodium, including glucose, phosphate, urate, and amino acids. The resulting constellation of abnormalities, the so-called Fanconi syndrome, was once common in heavily contaminated regions of Japan [82]. As this syndrome is now a rare manifestation of Cd tubulopathy, it is not considered in this review.

#### 4.3.1. Release of Intracellular Proteins into the Filtrate

After cellular injury, proteins synthesized in tubular cells may be released into the filtrate and detected in urine. In studies of Cd nephropathy, N-acetyl-β-D-glucosaminidase (NAG) and kidney injury molecule 1 (KIM1) have been assayed most frequently.

##### N-Acetyl-β-D-Glucosaminidase

NAG is present in lysosomes of proximal tubular cells. Mean normal rates of NAG excretion rise slightly with age [204]. The molecular weight of NAG, 150 kD, precludes glomerular filtration and, therefore, ensures that excreted NAG has emanated from tubules. The enzyme exists in two major isoforms, A and B. NAG-A is released by exocytosis into the filtrate at a stable rate that is unrelated to the cellular Cd content. NAG-B remains in lysosomes and enters the filtrate after cellular injury [204,205]. Its excretion is increased by Cd toxicity [205,206,207]. Reported measurements of NAG excretion have contributed significantly to our current synthesis of Cd nephropathy (see Section 4.3.2, Section 4.3.3, and Section 4.3.6).

##### Kidney Injury Molecule 1

KIM1 is a transmembrane glycoprotein that participates in the restoration of adhesion between regenerated proximal tubular cells. During this process, the ectodomain of KIM1 is released into the filtrate and excreted in urine. The protein is not detectable in the absence of injury [208].

KIM1 has been studied extensively in animals as a biomarker of Cd toxicity [209,210,211]. In rats treated with exogenous Cd, genetic expression and urinary excretion of KIM1 (E_KIM1_) increased at 6 weeks; E_Cd_ and E_NAG_ rose 3 and 6 weeks later [209,210]. Apoptosis was sparsely evident at 6 weeks and more prevalent at 12 weeks; increased KIM1 excretion accompanied apoptosis but was also observed in the absence of apoptosis [211]. If rodent studies can be extrapolated to humans, E_KIM1_ identifies toxicity that has not yet led to cell death, and it is detectable before E_Cd_ rises. It appears to be the earliest appearing indicator of Cd tubulopathy.

At least four studies have examined the utility of E_KIM1_ in humans exposed to Cd. Pennemans and colleagues investigated a Belgian sample with chronic low-dose environmental exposure [212]. The geometric mean of Cd excretion in this group was 0.76 μg/g creatinine, which is approximately twice that reported in healthy populations and 15% of the conventional Cd threshold for tubular injury (5.24 µg/g creatinine) [41]. Pennemans and colleagues found that E_KIM1_ correlated with E_Cd_ even though the excretion of reabsorptive markers did not. A Thai group confirmed this correlation in a sample with higher E_Cd_ [213], but a Chinese report did not [214]. A second study from China showed that E_KIM1_ rose in a stepwise fashion with low-, middle-, and high-dose environmental exposure to Cd [58].

#### 4.3.2. Excretion of Cadmium

Excreted Cd is either filtered and not reabsorbed or released from tubular cells into the filtrate [215]. Several lines of evidence support the second alternative. After inducing Cd nephropathy in rabbits, Nomiyama and Foulkes infused labelled CdMT at increasing rates to create a series of steady-state plasma concentrations of the complex. Although Cd poisoning had reduced the tubular maximum for CdMT, the excretion rates of total Cd greatly exceeded those accounted for by failure to reabsorb the label [216]. Most of the excreted Cd, therefore, emanated from the tubular cells.

In kidneys from rats intoxicated with Cd, Tanimoto and colleagues showed that E_Cd_ and numbers of apoptotic, sloughed cells in tubules rose in tandem [217]. In workers with occupational exposure to Cd, E_Cd_ correlated with the renal cortical content of the metal, as measured by neutron-capture gamma-ray analysis [218]. In human accident victims, the Cd content of renal tissue at autopsy correlated with the urine Cd concentration ([136]; Figure 2; see Section 4.1.1). In transplanted kidneys, the tissue Cd content correlated with the preoperative overnight E_Cd_ of living donors [140]. Three groups of investigators found that E_Cd_ varied directly with GFR, as though the number of intact nephrons had determined the rate of appearance in urine [219,220,221].

Reported correlations of E_Cd_ with E_NAG_ or E_KIM1_ provide additional evidence that urinary Cd emanates from tubular cells [205,212,214,222,223,224,225,226]. Importantly, these studies did not suggest that markers of cell injury began to rise at a threshold level of E_Cd_; instead, the correlations extended in a linear fashion from normal to increased levels of E_Cd_ [205,222,225,226]. E_KIM1_, the most sensitive indicator of Cd-induced toxicity, rose before E_Cd_ in animal studies [209,210].

Taken together, the foregoing observations suggest that Cd excretion does not result from a failure to reabsorb filtered Cd. It is more likely that Cd, NAG, and KIM1 emanate from the same source for the same reason. We infer that E_Cd_ is itself a marker of tubular cell injury.

#### 4.3.3. Cadmium Toxicity and the Glomerular Filtration Rate (GFR)

A paradox emerges from the literature relating E_Cd_ to GFR. Three groups have reported that E_Cd_ rose with GFR in exposed populations [219,220,221], and three have stated that C_cr_ or eGFR declined steadily as E_Cd_ increased from modest levels [51,52,227,228,229]. To reconcile these observations, we speculate that in the progression of Cd nephropathy, cellular injury is evident before nephrons are lost; during that phase, E_Cd_ varies directly with the nephron number, which, in turn, varies directly with GFR. Cell death ensues as the burden of Cd rises; during this phase, Cd is released to filtrate at an increased rate even though nephrons are disappearing simultaneously. Estimated GFR falls as E_Cd_ continues to rise.

In Thai population samples, our group found inverse relationships between eGFR and E_Cd_ at all levels of environmental Cd exposure [52,54]. Moreover, investigators from multiple geographic regions documented a progressive decline in GFR despite mitigation or termination of occupational exposure [229,230,231,232]. This decline may have resulted from the continued transfer of CdMT from the liver to the kidneys [233], or it may have reflected continuous nephron destruction by a stable renal burden of the metal [234,235].

#### 4.3.4. Impaired Reabsorption of Small Filterable Proteins

Small proteins are readily filtered by normal glomeruli and reabsorbed and degraded by proximal tubular cells. As reabsorption of such proteins is virtually complete, excretion rates above a cutoff value may be viewed as evidence of impaired reabsorptive capacity. The proteins most commonly studied for this purpose are β_2_-microglobulin (β_2_MG) and retinol-binding protein 4 (RBP4).

##### β2-Microglobulin

β_2_MG is the light chain of class I major histocompatibility complexes and is, therefore, found on most nucleated cells. Its molecular weight is 11,000 Daltons. The rate at which β_2_MG enters plasma (“influx”) is relatively constant within and among healthy subjects, but it may rise in patients with chronic inflammatory conditions or hematologic malignancies [236].

β_2_MG is eliminated exclusively by the kidneys. A modest fraction of the amount removed is taken up from peritubular capillaries [237], but most elimination results from glomerular filtration, proximal tubular reabsorption, and intracellular degradation. At least 90% of the circulating protein is ultrafilterable [238,239], and 99.9% of the filtered load is ordinarily reabsorbed. When the GFR is normal, the equilibrium between influx and renal processing establishes a plasma concentration ([β_2_MG]_p_) between 1.2 and 2.7 mg/L [236]. As GFR falls, the filtrate is presented to proximal tubules at a rate that is absolutely reduced but normal or increased per surviving nephron. [β_2_MG]_p_ rises secondarily, and equilibrium between the influx and the degradation of the protein is maintained [240,241,242,243,244].

In Cd research, it has been customary to declare that proximal tubular toxicity is present at E_β2MG_ > 300 µg/g creatinine [41]. At an arbitrary E_β2MG_ of 300 µg/d, E_cr_ of 1 g/d, GFR of 144 L/d (100 mL/min), and filterable [β_2_MG]_p_ of 2.0 mg/L, fractional excretion of β_2_MG (FE_β2MG_) is 0.1% and fractional reabsorption (FR_β2MG_) is 99.9%. Doubling of E_β2MG_ to 600 µg/g creatinine, a clearly elevated value, entails an increase in FE_β2MG_ from 0.1% to 0.2% and a reduction in FR_β2MG_ to 99.8%. Miniscule Cd-induced reductions in FR_β2MG_, therefore, lead to substantial increments in E_β2MG_ [245].

The sensitivity of E_β2MG_ to slight reductions of FR_β2MG_ should not be interpreted as evidence that the underlying cellular injury is trivial. Values of E_Cd_ at which E_β2MG_ exceeds 300µg/g creatinine are at least 10 times higher than in normal populations [246,247]. If E_Cd_ itself is a marker of toxicity, then the customary cutoff value of E_β2MG_ is not a sensitive metric for detecting tubular injury. For pathophysiologic insight, E_β2MG_ is most logically related to the normal *maximal* reabsorptive capacity for the protein—i.e., the tubular maximum (Tm_β2MG_)—if such a Tm exists. Hall could not demonstrate one in dogs with an infusion of human β_2_MG [237], but in rats, Gauthier documented a Tm_β2MG_ when [β_2_MG]_p_ was approximately four times the norm [238].

In theory, if a Tm_β2MG_ existed in humans, a decline in GFR might expose it. In this circumstance, surviving nephrons would be presented with a higher concentration of β_2_MG in less total filtrate volume, and a normal rate of presentation to a reduced nephron mass could exceed a putative Tm_β2MG_. Multiple investigators have argued that this scenario occurs, but it is often possible that the disease lowering GFR has also lowered Tm_β2MG_ [241,244]. In patients with hepatorenal syndrome, in which the perfusion of normal kidneys is severely limited, a Tm_β2MG_ was not demonstrable despite extreme reductions in GFR and elevations in [β_2_MG]_p_ [243]. Similarly, in children with glomerular disease exclusively, on biopsy, FE_β2MG_ did not correlate with GFR [244].

If some humans can reabsorb all filtered β_2_MG despite a low GFR and high [β_2_MG]_p_, then nephron loss is insufficient to explain excessive E_β2MG_ in patients with Cd nephropathy. It appears that Cd imposes a Tm_β2MG_ or reduces one that already exists, and increased E_β2MG_ indicates reduced β_2_MG reabsorption per nephron at any GFR [248]. Once Cd has established a Tm_β2MG_, we expect E_β2MG_ to rise substantially as GFR falls. Multiple investigators have documented this phenomenon [53,232,249], but none have quantified the individual contributions of GFR and Tm_β2MG_ to excessive E_β2MG_.

##### Retinol-Binding Protein 4

RBP4, a small protein with molecular weight 21,000 Daltons, is synthesized in the liver. As its name implies, it binds to retinol (vitamin A) and transports the vitamin to tissues. Most RBP4 in plasma is bound to transthyretin (pre-albumin) in a complex that is too large to be filtered by normal glomeruli, but a minor fraction is unbound and readily filtered [239,250]. The total plasma concentration of RBP4 ([RBP]_p_) is normally 40–60 µg/mL [251].

As is the case with β_2_MG, over 99.9% of filtered RBP is normally reabsorbed. Urinary excretion (E_RBP_) increases markedly in tubulointerstitial disease, and it also rises substantially as GFR falls [233,252]. We are unable to ascertain whether nephron loss per se raises free [RBP]_p_ to a threshold at which the reabsorptive capacity of surviving nephrons is exceeded. Bernard and colleagues reported a threshold [RBP]_p_ of 25 mg/L at which [RBP]_u_ rose acutely, but all of the patients with CKD had tubulopathies [253]. Mason and colleagues described a similar finding at high rates of Cd excretion, but we presume that their subjects had sustained Cd-induced tubular injury [233].

RBP4 differs in some respects from β_2_MG. β_2_MG is a positive acute-phase reactant (APR), and its plasma concentration rises in association with inflammation. RBP4 is a negative APR, and its concentration falls with inflammation [250]. Whereas β_2_MG is unstable at pH < 5.5, RBP4 is stable at any physiologic urine pH. Because of this attribute, some have argued that RBP4 should be the reabsorptive marker of choice in studies of Cd tubulopathy [253].

#### 4.3.5. Normalization of Excretion Rates to Creatinine Excretion or Creatinine Clearance

The kidney is the final repository of assimilated Cd and the principal site of persistent toxicity [254]. To study that toxicity, it is reasonable to quantify excretion rates of relevant substances (abbreviated E*_x_* for a given substance *x*). In practice, however, urine aliquots are more conveniently obtained than timed collections, and concentrations of *x* ([*x*]_u_) are measured instead of E*_x_*. To nullify the effect of urine volume on these concentrations, [*x*]_u_ is usually normalized to the urine creatinine concentration ([cr]_u_) because volume affects [*x*]_u_ and [cr]_u_ proportionately.

This practice may lead to erroneous conclusions. As E*_x_* and E_cr_ are biologically unrelated, each excretion rate is influenced by at least one variable that does not affect the other. E_cr_ is determined primarily by muscle mass [255], which has no relationship to E*_x_*; consequently, in a physically diverse population, normalization of a given [*x*]_u_ to [cr]_u_ alters [*x*]_u_/[cr]_u_—in theory, by as much as fourfold—for a reason unrelated to E*_x_* [256]. Conversely, if substance *x* emanates from tubular cells, E*_x_* varies directly with the number of cells and the intracellular concentration of *x*, neither of which is related to E_cr_. If nephron mass is normal, E*_x_* may rise as a consequence of cellular injury; if Cd destroys nephrons, E*_x_* may fall. As E_cr_ does not change importantly in either circumstance, [*x*]_u_/[cr]_u_ may overstate tubular injury per nephron when GFR is normal and understate it when GFR is reduced [221,229].

To circumvent these issues, we recently introduced the practice of normalizing E*_x_* to creatinine clearance (C_cr_), a surrogate for GFR, in studies of Cd nephrotoxicity [52,54]. C_cr_ is the excretion rate divided by the plasma concentration of creatinine; if V_u_ is the urine flow rate, C_cr_ = [cr]_u_V_u_/[cr]_p_ and E*_x_*/C_cr_ = [*x*]_u_V_u_/([cr]_u_V_u_/[cr]_p_), which simplifies to [*x*]_u_[cr]_p_/[cr]_u_. Whereas the unit of [*x*]_u_/[cr]_u_ is mass of *x* per mass of creatinine, the unit of E*_x_*/C_cr_ is mass of *x* excreted *per volume of filtrate*. Since C_cr_ varies directly with the number of nephrons, E*_x_*/C_cr_ also depicts excretion of *x* per intact nephron. As the formula for E*_x_*/C_cr_ includes the ratio [*x*]_u_/[cr]_u_, E*_x_*/C_cr_, like [*x*]_u_/[cr]_u_, is unaffected by urine volume; since [cr]_p_ rises with E_cr_ at a given C_cr_, E*_x_*/C_cr_ is also unaffected by muscle mass. Most importantly, if substance *x* is released by tubular cells into urine, E*_x_*/C_cr_ prevents overstatement of injury per nephron at normal GFR and understatement at reduced GFR.

#### 4.3.6. A Pathophysiologic Synopsis of Cadmium Nephropathy

We recently published an analysis of cross-sectional data from Thai subjects living in areas of low, moderate, and high intensity of environmental Cd exposure. The patients were clinically well and were not hemodynamically predisposed to reductions in GFR [54]. In each subset and in the entire sample, we examined linear and quadratic regressions of eGFR on E_Cd_/C_cr_ and E_NAG_/C_cr_, regressions of E_NAG_/C_cr_ on E_Cd_/C_cr_, and regressions of E_β2MG_ on E_Cd_/C_cr_ and E_NAG_/C_cr_. All regressions were statistically significant except those of E_β2MG_/C_cr_ on E_Cd_/C_cr_ and E_NAG_/C_cr_ in the low-exposure subset. In general, effect size (standardized β) and coefficients of determination (R^2^) rose with exposure intensity. A minority of subjects was found to have eGFR < 60 mL/min/1.73 m^2^; in the absence of renal hypoperfusion, which would have been accompanied by disqualifying signs and symptoms, the only plausible explanation for subnormal glomerular filtration was a reduction in the number of intact nephrons. “Nephron loss” is a widely used term to describe this state [257].

Our goals in the analysis of these data were to explain the correlation of E_Cd_/C_cr_ with E_NAG_/C_cr_, identify the source of excreted Cd, and elucidate the inverse relationship of eGFR to E_Cd_/C_cr_ and E_NAG_/C_cr_. Although we recognized that tubular injury might interfere with reabsorption of filtered CdMT, we doubted that *this interference* would lead to a statistically significant relationship of E_NAG_/C_cr_ to E_Cd_/C_cr_. As multiple lines of evidence suggested that excreted Cd, like NAG, emanates from proximal tubular cells (see Section 4.3.2), we reasoned that a common origin of the two substances would account for the relationship between E_NAG_ and E_Cd_.

Whereas we consider both E_Cd_/C_cr_ and E_NAG_/C_cr_ to be parameters of cellular injury at the time of testing, eGFR reflects progressive nephron loss due to continuous accrual of Cd in proximal tubules. To explain why eGFR varied inversely with E_Cd_/C_cr_ and E_NAG_/C_cr_ despite these temporal differences, we argued that all three parameters were either current or historical functions of the same intracellular Cd content. We concluded that Cd-induced injury had led to tubular cell death and a reduction of GFR. Relationships of eGFR to E_Cd_/C_cr_ and E_NAG_/C_cr_ suggested that the severity of cellular injury had determined the extent of nephron loss.

In the literature on Cd toxicity, we occasionally encounter the concept that a reduction of GFR implies injury to glomeruli by the metal. Ample evidence suggests that this concept is both erroneous and unnecessary. CKD is a common sequela of ischemic acute tubular necrosis and numerous acute and chronic tubulointerstitial (TI) diseases that do not affect glomeruli [257,258,259,260,261,262]. Moreover, primary glomerular disease also leads to TI inflammation and fibrosis, presumably because reabsorbed, inappropriately filtered proteins are toxic to tubular cells [263]. Whether glomeruli or tubules are injured initially, the extent of TI fibrosis is the histologic finding that correlates best with GFR in CKD [264,265]. Possible filtration-reducing effects of TI fibrosis include the destruction of post-glomerular peritubular capillaries, amputation of glomeruli from tubules, and obstruction of nephrons with cellular debris [257,266].

We have not found English-language reports relating histopathology to GFR in asymptomatic humans exposed to Cd. However, in 61 autopsied subjects with itai-itai disease (IID), a syndrome of painful osteomalacia and proximal tubular dysfunction associated with severe Cd toxicity, Baba and colleagues showed that the most extreme osteomalacia was associated with the most advanced renal shrinkage [267]. In autopsies of 15 patients with IID, Yasuda and colleagues found that low kidney weight correlated with loss of tubules on microscopy; severely afflicted kidneys showed interstitial fibrosis and widespread atrophy of tubular epithelium [268]. Although reduced kidney weight and disrupted cortical architecture suggest that GFRs were reduced in these studies, neither Baba nor Yasuda provided relevant quantitative information. In contrast, Saito and colleagues performed extensive renal function studies (but no histopathology) in 13 patients with IID; endogenous creatinine clearance, a surrogate for GFR, was reduced in 12 [269]. Nogawa and associates measured serum creatinine concentrations in 4 of 5 patients with severe skeletal manifestations of IID, and the concentrations were substantially increased in each case [270]. Yasuda mentions that numerous Japanese patients with IID required chronic dialysis [268]; this choice of treatment suggests that extreme Cd toxicity reduced the GFR to levels that could not sustain life.

#### 4.3.7. Assessment of Cadmium Nephrotoxicity: Summary

Cd is a cumulative toxin to proximal tubular cells. Ample evidence suggests that the metal inflicts injury by promoting the creation of reactive oxygen species. The injury commences at a low intracellular concentration of Cd and intensifies as the concentration rises. Excretion of KIM1 is the first identifiable manifestation of toxicity, and NAG and Cd are subsequently released from injured or apoptotic cells. Inflammation and fibrosis follow, nephrons are lost, and GFR falls. After significant proximal tubular injury has occurred, reabsorption of small filtered proteins decreases and excretion of these proteins exceeds the normal limit. Once a Tm_β2MG_ is established, E_β2MG_ rises rapidly as GFR falls.

E_NAG_ and E_KIM1_ correlate with E_Cd_. This observation and many others suggest that Cd excretion results from the cellular release of the metal rather than filtration without reabsorption. If this conclusion is accepted, then increased E_Cd_ is itself a manifestation of Cd toxicity, and the concept of a threshold E_Cd_ at which E_β2MG_ becomes excessive loses its pathophysiologic relevance. In subjects with low environmental exposure to Cd, eGFR is statistically related to E_Cd_/C_cr_ when E_β2MG_/C_cr_ is not, and the relationship grows stronger as exposure increases. Markers of cellular injury at the time of testing, e.g., E_NAG_/C_cr_ and E_Cd_/C_cr_, correlate with eGFR, an indicator of historical injury, because all three parameters are determined by the intracellular concentration of Cd. Tubular injury inflicted by Cd is sufficient to explain reductions in GFR and progression of CKD.

## 5. Environmental Exposure to Cd and Pb, Toxic Kidney Burden, CKD, and Other Common Ailments

Environmental exposures are estimated to account for 70–90% of the risk of acquiring chronic ailments such as diabetes type 2, CKD and cancer [271,272]. The kidney is particularly at risk of injury from long-term use of therapeutic drugs and chronic exposure to environmental toxicants, especially when they are present in the diet [273,274,275]. The increased risk of kidney injury is attributed to its large blood flow (20–25% of cardiac output) and exposure to high solute concentrations as the primary glomerular filtrate is concentrated [276]. In the following sections, we discuss cross-sectional studies that suggest that Cd and Pb are synergistic CKD risk factors and longitudinal studies that implicate combined Cd and Pb exposure in enhanced mortality risk. 

### 5.1. The Increased Risk of CKD Associated with Cadmium and Lead Exposure

CKD afflicts 8% to 16% of the world population. Diabetes and hypertension are the most common risk factors universally, while obesity is an additional risk factor, especially in industrialized countries [272,273,274,275,276,277,278,279]. CKD is a cause of morbidity and mortality as it is an important predictor of end-stage kidney disease (ESKD), stroke and cardiovascular disease (CVD) [280,281,282,283,284,285]. CKD is characterized by albuminuria (a urinary albumin to creatinine ratio, uACR, above 30 μg/g) and/or a decrease of GFR to ≤60 mL/min/1.73 m^2^ that persists for at least three months [48,49,50].

GFR is considered the best indicator of overall kidney function because it reflects the number of functioning nephrons at any given time [50]. In practice, the GFR is estimated from equations, notably, the Chronic Kidney Disease Epidemiology Collaboration (CKD-EPI) equations [47,48,49], and is reported as eGFR. The CKD-EPI equations, which have been validated using inulin clearance, are considered as the most accurate approximation of GFR [286]. CKD in its early stage is asymptomatic, and CKD staging is vital to evaluate nephron loss. Accordingly, CKD stages 1, 2, 3a, 3b, 4, and 5 correspond to eGFR of 90–119, 60–89, 45–59, 30−44, 15–29, and <15 mL/min/1.73 m^2^, respectively [48,287]. For simplicity, a low eGFR refers to an eGFR of <60 mL/min/1.73 m^2^, and albuminuria refers to uACRs above 30 μg/g.

#### 5.1.1. U.S. Population

An increment of [Pb]_b_ to 10 μg/dL was associated with a decrease in creatinine clearance of 10.4 mL/min in an early study of U.S. men participating in the Normative Aging Study between 1988 and 1991 [288]. Subsequently, an increased risk of CKD was associated with Pb and Cd exposures in participants of various NHANES cycles. In NHANES 1999–2006, adults with [Cd]_u_ levels ≥ 1 µg/L had 1.48- and 1.41-fold increases in the risk of low eGFR and albuminuria [44], while those with [Cd]_b_ ≥0.6 μg/L had 1.53-, 1.92-, and 2.91-fold increases in the risk of low eGFR, albuminuria and low eGFR plus albuminuria, respectively [289]. In addition, [Pb]_b_ ≥ 2.4 μg/dL, which is 12% of the exposure limit in occupational exposure settings of 20 μg/dL, was associated with a 1.56-fold increase in the risk of low eGFR [289]. Of interest, the risk of CKD was increased further when subjects were exposed to both metals: the odds ratios for low eGFR, albuminuria, low eGFR plus albuminuria rose to 1.98, 2.34, and 4.10 in participants who had both [Cd]_b_ and [Pb]_b_ in the highest quartiles, compared with those who had [Cd]_b_ and [Pb]_b_ in the lowest quartiles [289]. Likewise, in adults enrolled in NHANES 2007–2012, [Cd]_b_ > 0.61 μg/L was associated, respectively, with 1.80- and 1.60-fold higher risk of having low eGFR and albuminuria, compared with [Cd]_b_ ≤ 0.11 μg/L [45]. A pronounced effect of Cd on eGFR was seen in women who had diabetes and/or hypertension. On average, women with diabetes, hypertension and [Cd]_b_ in the highest quartile (0.61−9.3 μg/L) had 4.9 mL/min/1.73 m^2^ lower eGFR than nondiabetic, normotensive women who had the lowest [Cd]_b_ (0.11−0.21 μg/L) [46]. In those women with hypertension and the highest [Cd]_b_ quartile, the mean eGFR was 5.77 mL/min/1.73 m^2^ lower than the normotensive with the same lowest [Cd]_b_ quartile [46].

In another analysis of data from adult participants in NHANES 2011–2012, [Cd]_b_ > 0.53 μg/L was associated with 2.21- and 2.04-fold increases in the risk of low eGFR and albuminuria, respectively. [Cd]_u_ as low as 0.22 μg/L was associated with higher urinary albumin excretion, compared with [Cd]_u_ < 0.126 μg/L, but neither Pb nor Hg was associated with elevated albumin excretion [290].

#### 5.1.2. Swedish Population

In a study of Swedish women, 53–64 years of age, [Cd]_u_ ≥ 0.6 μg/g creatinine was associated with a significant increase in tubular injury and decrease in eGFR [227]. In a longitudinal study (*n* = 4341), [Pb]_b_ ≥ 3.3 μg/dL was associated with a 1.49-fold increase in the incidence of CKD, and the mean eGFR of subjects with this range of [Pb]_b_ fell by 24 mL/min/1.73 m^2^ during a 16-year follow-up period [291]. In a prospective, nested case–control study, 118 cohort participants developed ESKD during a 7-year period [292]. The mean values for erythrocyte Cd and Pb of these cases were 1.3 and 7.6 μg/dL, respectively. After adjusting for potential confounders, including Cd and Hg, smoking, body mass index, diabetes, and hypertension, only erythrocyte Pb was associated with an increase in the risk of developing ESKD [292].

#### 5.1.3. Thai Population

Even though the environmental exposure of the general population in Thailand to Cd and Pb is low [293], a Bangkok study has shown that even a urinary Cd excretion rate (E_Cd_) as low as 0.38 μg/L (0.44 μg/g creatinine) was associated with a decrease in eGFR [12]. The risk of a decrease in eGFR was 2.9-fold higher in those who had E_Cd_ in the highest quartile. Likewise, in those with [Pb]_u_ in the highest quartile, the risk of eGFR decrease was 2.3-fold higher than those in the lowest quartile of [Pb]_u_ [12]. Of note, a positive association between eGFR levels and serum ferritin in men suggested a protective effect of adequate body iron status. Women may be more predisposed to absorption of ingested Cd and Pb because of their lower levels of body iron stores [12].

In a Cd-polluted region of Thailand, more than half (66%) of the residents had elevated Cd body burdens, reflected by E_Cd_ ≥ 2 μg/g creatinine, and the prevalence of CKD was 16.1% [294]. In a 5-year follow up study, a further decrease in eGFR was observed in residents who had high Cd exposure (E_Cd_ ≥ 5 μg/g creatinine) [229]. These findings suggest that nephron loss associated with high Cd exposure and increasing kidney dysfunction continues even when the consumption of Cd-contaminated rice is reduced [229]. Other studies have reported an even greater effect of Cd on eGFR in women who had hypertension [51,52], as has been noted in the U.S. population study [46].

#### 5.1.4. Chinese Population

In a Chinese population study (*n* = 8429), intake levels of dietary Cd were inversely associated with eGFR, and the risk of CKD rose with Cd intake levels in a dose-dependent manner: Cd intake levels of 23.2, 29.6 and 36.9 μg/day were associated with 1.73-, 2.93- and 4.05-fold increments of CKD risk, compared with a Cd intake level of 16.7 μg/day [15]. [Pb]_b_ of 10 μg/dL was associated with tubular injury in Chinese men who were exposed to Pb in the workplace [295]. In a coexposure analysis of residents in Cd-polluted and control areas in China [296], the risk of tubular injury, as assessed with [NAG]_u_, was highest in subjects who had [Cd]_b_ ≥ 2 μg/L and [Pb]_b_ ≥ 10 μg/dL, while the risk of a decrease in eGFR was highest in those with E_Cd_ ≥ 3 μg/g creatinine and E_Pb_ ≥ 10 μg/g creatinine.

#### 5.1.5. Korean and Belgian Populations

The prevalence of CKD in a representative Korean population (*n* = 1797) was 7.1% and the population means for [Pb]_b_, [Hg]_b_ and [Cd]_b_ were 2.37, 4.35 and 1.17 μg/L, respectively [297]. Elevated [Cd]_b_ levels were associated with 1.52- and 1.92-fold increases in the risk of CKD in those with diabetes and hypertension, respectively. Neither [Pb]_b_ nor [Hg]_b_ showed such a relationship [297]. Supporting Cd as a risk factor for CKD is another study of 2992 Koreans, 20–65 years of age; [Cd]_b_ > 1.74 μg/L was associated with a 1.97-fold increase in odds for CKD in women [298]. In a study of a subset of participants (*n* = 2005, aged ≥20 years) in a nationwide survey (*n* = 8641), Cd exposure was again found to be an important risk factor for CKD in Korea: [Cd]_b_ levels in the highest quartile (mean, 2.08 μg/L) were associated with a 1.93-fold increase in the risk of CKD [283]. In contrast, [Pb]_b_ levels in the highest quartile (mean, 4.13 μg/dl) were not associated with a significant increase in CKD risk in this subset analysis [299].

Intriguingly, although Pb exposure levels in Korea did not seem to affect CKD risk, evidence that Pb coexposure may enhance Cd toxicity in kidneys has emerged from another Korean population study (*n* = 1953, aged 18–83 years) in which [Pb]_b_ and [Cd]_u_ both correlated positively with [β_2_MG]_u_, a marker of tubular dysfunction [300]. However, the correlation between [Cd]_b_ and [β_2_MG]_u_ was strengthened in those who had [Pb]_b_ above the median of 2.20 μg/dL. Similar to Korean findings, a study of Belgian metallurgic refinery workers suggested that there is a Cd–Pb interaction [301]. The associations between [Cd]_u_, [NAG]_u_ and [RBP]_u_ were only seen in workers who had high levels of [Pb]_b_ (≥21.9 μg/dL), corresponding to the 75th percentile or higher. In addition, the associations between [Cd]_b_, [NAG]_u_ and [intestinal alkaline phosphatase]_u_ only became statistically significant in workers who had [Pb]_b_ ≥ 21.9 μg/dL [285]. This Cd–Pb interaction was seen although blood Pb in Belgian workers of 21.9 μg/dL was 6-fold higher than the 90th percentile blood Pb level of 3.66 μg/dL in a Korean study [300]. Of note, [Pb]_b_ of ~20 μg/dL did not exceed the exposure limit for neurotoxicity in adults of 25 μg/dL [71]. Further epidemiologic research is required to examine the mechanisms underlying the interaction of Cd and Pb in chronic Cd and Pb exposure conditions.

### 5.2. Environmental Exposures and Mortality from All Causes

In the previous section, exposures to Cd and Pb have been identified as risk factors for CKD across populations. In this section, we summarize the observations across populations that demonstrated the overall impact of chronic lifelong exposure to Cd and Pb on life prognosis and risks of death from CVD and cancer.

#### 5.2.1. Cadmium and Mortality in the U.S.

Temporal trend analysis indicated a 29% reduction in environmental Cd exposure among a representative cohort of men in the U.S. over an 18-year follow-up period (1988–2006) during which the mean E_Cd_ fell from 0.58 to 0.41 μg/g creatinine [302]. A reduction in environmental Cd exposure in women over the same 18-year period was statistically insignificant.

Cd exposure was associated with heart disease in cross-sectional studies [303,304,305]. Cd exposure was also an independent risk factor for ischemic stroke in another cross-sectional study (*n* = 2540), with a mean and a 75th percentile E_Cd_ of 0.42 and 0.68 μg/g creatinine, respectively [306]. These associations may account for the increased mortality from CVD seen in various follow-up studies of NHANES participants. In a follow-up study of participants in NHANES 1988–2006, [Cd]_b_ was linked to an increase in death from CVD, especially in women [302]. E_Cd_ of ≥0.37 to ≥0.65 μg/g creatinine was linked to increased risk of death from heart disease among participants in NHANES 1999–2008 [307,308].

A 4.29-fold increase in death from malignant disease was seen among participants from NHANES (1988–1994) who had E_Cd_ > 0.48 µg/g creatinine [309]. In men only, a 2-fold increase in E_Cd_ was respectively associated with 28%, 55%, 21%, and 36% increases in death from all causes, cancer, CVD, and coronary heart disease, after adjustment for potential confounders, including cigarette smoking [309]. E_Cd_ of ≥0.37 to ≥0.65 μg/g creatinine was linked to increased risk of breast cancer among women participating in NHANES 1999–2008 [307,308].

In other follow-up studies of NHANES 1988–1994 participants, a two-fold increase in E_Cd_ was associated with 26% and 21% increases in cancer mortality in men and women, respectively [310]. The mortality from lung cancer in men was increased by 3.22-fold, while the mortality from liver-related nonmalignant disease was increased by 3.42-fold in participants who had E_Cd_ of ≥0.58 to ≥0.65 μg/g creatinine [310,311].

Among the ≥65 years of age participants of NHANES 1999–2004, [Cd]_b_ levels > 0.6 μg/L were associated with a 3.83-fold increase in the risk of the mortality from Alzheimer’s disease [312]. In another publication based on the data from the same NHANES cycle showed that elevated urinary Cd levels were associated with a 58% increase in the risk of death from Alzheimer’s disease in the 60−85 years age group [313].

#### 5.2.2. Cadmium and Mortality in Sweden and Australia

In a Swedish cohort study, the overall mortality was increased by 2.06-fold in women who had [Cd]_b_ ≥ 0.69 μg/L compared with those with [Cd]_b_ ≤ 0.18 μg/L [314]. In this Swedish study on women, the median, 25th and 75th percentile levels of [Cd]_b_ were 0.28, 0.18 and 0.51 μg/L, respectively [314]. In a follow-up study of women in Western Australia (*n* = 1359), there was 2.7-fold higher [Cd]_u_ in those with atherosclerotic vascular disease. This [Cd]_u_ was associated with a 36% increase in the risk of dying from heart failure and a 17% increase in the risk of having a heart failure event [299]. Hence, a reduction in survival was observed even though the kidney burden of Cd among the study women was low: the median, 25th and 75th percentile levels of [Cd]_u_ were 0.18, 0.09 and 0.32 μg/L, respectively [315].

#### 5.2.3. Cadmium and Mortality in Japan

A dose–response relationship between mortality risk and elevated body burden of Cd was seen in men who were residents of nonpolluted areas of Japan: the mortality risk from all causes was increased by 35% and 64% in men who had E_Cd_ 1.96–3.22 and ≥3.23 µg/g creatinine, respectively [316]. In women from the same nonpolluted areas, a 49% increase in mortality from all causes was associated with E_Cd_ ≥ 4.66 µg/g creatinine. A 6% increase risk of death from cancer at any site was also seen only in women. There was a 13% increase in mortality from pancreatic cancer for every 1 μg/g creatinine increment of E_Cd_ [317].

In a region of Japan with Cd pollution, all-cause mortality increased by 1.57-and 2.40-fold in men and women with proteinuria and glycosuria, attributable to their elevated Cd exposure [318]. An increase in deaths from ischemic heart disease and incidences of diabetes and kidney disease was observed [302]. A 1.49-fold increase in deaths from cancer in any site was observed, especially in women with evidence of Cd-related kidney pathologies [319]. The increase in the risk of dying from a specific cancer type was 3.85, 7.71 and 10.1 for cancer of the uterus, kidney and kidney plus urinary tract [319]. The median [Cd]_u_ in women and men with proteinuria and glycosuria was 8.3 μg/L and 10 μg/L, respectively. Paradoxically, in men, the risk of lung cancer and the risk of dying from cancer were reduced by 47% and 21%, respectively [319].

#### 5.2.4. Lead and Mortality in the U.S., Korea and China

In a follow-up study of a subset of participants in NHANES 1988–1994, there was a 48% increase in cancer mortality risk in those with [Pb]_b_ ≥ 5 μg/dL [320]. In another follow-up of participants in the same cycle, [Pb]_b_ 1.0–6.7 μg/dL was associated with 1.37-, 1.70- and 2.08-fold increases in death from all causes, CVD, and ischemic heart disease [321]. In the NHANES 1999–2010 follow-up study (*n* = 5316), exposure to low levels of Pb, reflected by [Pb]_u_ > 1.26 μg/dL, may increase the risk of deaths from all causes and cancer by 1.79- and 6.60-fold, respectively [196]. A 44% increase in CVD mortality was observed for every 10-fold increase in hematocrit-corrected [Pb]_b_ in a follow-up of participants, aged ≥40 years, in NHANES 1999–2010 (*n* = 18,602) [322].

In a cohort study of lead-exposed workers of South Korea (*n* = 81,067), [Pb]_b_ 10–20 μg/dL was associated with 36% and 93% increases in the risk of death from all causes in men and women, respectively [323]. The mortality risk from bronchial and lung cancer rose by 10.45- and 12.68-fold in female workers with [Pb]_b_ of 10–20 μg/dL. In male workers, the same [Pb]_b_ range of 10–20 μg/dL was associated with hospital admission for ischemic heart disease, cerebrovascular disease, angina pectoris and cerebral infarction [324].

A 25% increase in deaths from all causes was recorded in a follow-up study of a Chinese population (*n* = 2832) with the median Pb intake level of 101.9 μg/day [16]. Compared with Pb intake levels in Quartile 1 (67 μg/day), Pb intake levels in Quartile 3 (111.4 μg/day) and Quartile 4 (147 μg/day) were associated with 1.52- and 3-fold increases in cancer mortality, respectively [16].

Table 2 summarizes exposure levels reflected by blood concentrations, urinary excretion, and dietary intake estimates of Cd and Pb that have been associated with nephrotoxicity, enhanced risks for CKD, and mortality in various populations that include the U.S., Sweden, Australia, Japan, China, Thailand, Belgium, and Korea.

## 6. Summary and Conclusions

Dietary assessment by the total diet study method shows that both Cd and Pb are present in virtually all foodstuffs. Foods which are frequently consumed in large quantities, such as cereals, rice, potatoes and vegetables, contribute the most to the total intake of these toxic metals. Seafood (shellfish), offal, spinach, lettuce and chocolate are Cd sources among high consumers of these foods. Beverage, chocolate syrup, raisins, fish, meats (offal included), preserved soybean, and fungus products are sources of Pb for high consumers of these products. Cd intake levels of 23.2, 29.6 and 36.9 μg/day were associated with 1.73-, 2.93- and 4.05-fold increments of CKD risk, compared with the 16.7 μg/day intake rate. A Cd intake level of 23.2 µg/day is 40% of the FAO/WHO current tolerable intake level. Pb intake levels of 111.4 and 147 μg/day were associated with 1.52- and 3-fold increases in cancer mortality, compared with the 67 µg/day intake rate. A Pb intake level of 111.4 µg/day exceeds the FDA interim safe intake rate of 12.5 μg/day.

Historically, the health risk assessment of Cd has relied on E_β2MG_. This practice follows the FAO/WHO guidelines in which E_β2MG_ ≥ 300 μg/g creatinine were cutoff values to define the level of health concern (nephrotoxicity). However, multiple lines of evidence discussed in this review indicate that the established cutoff value of E_β2MG_ is not a sensitive indicator of tubular cell toxicity. KIM1 is the first identifiable marker of Cd-induced injury, and in our opinion, any elevation of E_Cd_ also signifies such injury. Estimated GFR is a function of intact nephron mass and is universally employed for diagnosis and staging of CKD. Health risk assessment of Cd should be based on the dose–response relationship between E_Cd_ and GFR.

The variable effect of low-level environmental exposure to Cd and Pb on GFR has caused some controversy. Consequently, governments worldwide have not established the necessary regulations to protect their populations. To improve comparability of guidelines among populations, normalization of [Cd]_u_ to C_cr_ is proposed to nullify urine flow rate as a confounder, circumvent the effect of muscle mass on [cr]_u_, and facilitate the expression of relevant excretion rates as functions of intact nephron mass.

Risk assessment of Cd is conventionally based on the urinary Cd threshold limit of 5.24 μg/g creatinine, which was the mean E_Cd_ at which E_ß2MG_ exceeded 300 µg/g creatinine. However, a [Cd]_u_ level as low as 1 μg/L (E_Cd_ ~0.5 μg/g creatinine) is associated with a significant increase in the risk of CKD and mortality from cardiovascular disease and cancer. As Cd and Pb exposure is highly prevalent, even a small increase in disease risk can result in a large number of people affected by a disease that is preventable. Environmental exposure to low-level Pb ([Pb]_b_ 1.0−6.7 μg/dL) is associated with mortality from cardiovascular disease and ischemic heart disease. [Pb]_u_ levels > 1.26 μg/L are associated with increased mortality from cancer.

Given the continuing rise in the incidence of CKD worldwide and the escalating treatment costs associated with dialysis and/or kidney transplants needed for survival, developing strategies to prevent CKD is of global importance. Furthermore, Cd and Pb are associated with cardiovascular morbidity and reduced life expectancy, independently of CKD. Prevention of Cd- and Pb-related ailments and mortality requires minimization of their environmental contamination. Accordingly, public measures to reduce environmental pollution and the food-chain transfer of Cd and Pb are vital, as are risk reduction measures through setting a maximally permissible concentration of Cd and Pb in staple foods to the lowest achievable levels.

## Figures and Tables

**Table 1:** Estimated intake levels of cadmium and lead and their sources.

Countries	Estimated Intake Levels as μg Per Day and Dietary Sources	
	Cadmium (Atomic Weight 112.4)	Lead (Atomic Weight 207.2)
China [89,90] 67% of population	Average consumers: 32.7 μg/day. Rice and vegetables as the main sources for most Chinese. Potato was the main source in Mongolia. High Cd foods: Nori, peanuts, squid, cuttlefish, and mushrooms.	Average consumers: 35.1 μg/day. Cereals, meats, vegetables, and beverages and water together contributed to 73.26% of total intake. High Pb foods: Kelp, nori, processed and preserved soybean, meat, and fungus. products.
Korea [92] *n* = 4867	Average consumers: 12.6 μg/day. Sources: Grain and grain-based products (40.4%), vegetables and vegetable products (16.5%), and fish and shellfish (17.9%). High Cd foods: Seaweed, shellfish and crustaceans, molluscs, nuts and seeds, and flavourings, with median values of 594, 186, 155, 15.7, and 6.23 μg/kg, respectively.	Average consumers: 9.8 μg/day. High Pb foods: Seaweed, shellfish and crustaceans, molluscs, fish, and sugar and sugar products, with respective median values of 94.2, 91.4, 62.4, 8.13, and 4.61 μg/kg, while the median value for beverages (fruit juice, carbonated fruit juice, carbonated drinks, sports drinks, and coffee) was 11.0 μg/kg.
Germany [94,100] *n* = 15,371	Average consumers: 14.6 μg/day. High consumers: 23.5 μg/day. Sources: Cereals and vegetables, beverages, fruits and nuts, and dairy products (milk included). High Cd foods: Cereals, oily seeds and fruits, and vegetables.	Average consumers: 37.1 μg/day. High consumers: 50.4 μg/day. Sources: Beverages, vegetables, fruits and nuts and cereals. High Pb foods: Meat (offal included), fish (seafood), vegetables and cereals.
Spain [95] *n* = 1281	Average consumers: 7.7 μg/day. Sources: Cereals and fish contributed to 38% and 29% of total intake. High Cd foods: Cereals (16.25 μg/kg), fish group (11.40 μg/kg).	Average consumers: 14.7 μg/day. Cereals contributed to 49% of total intake. High Pb foods: Sweeteners and condiments, vegetable oils, meat, and fish, with respective median levels of 32.5, 15.25, 14.90 and 13.21 μg/kg.
U.S. [84,97] *n* = 14,614 FDA 2014–2016 total diet study	Average consumers: 4.63 μg/day. Sources: Cereals and bread, leafy vegetables, potatoes, legumes and nuts, stem/root vegetables, and fruits contributed to 34%, 20%, 11%, 7% and 6% of total intake, respectively. High Cd foods: Spaghetti, bread, potatoes and potato chips contributed the most to total Cd intake, followed by lettuce, spinach, tomatoes, and beer. Lettuce was a main Cd source for whites and blacks. Tortillas and rice were main Cd sources for Hispanic Americans, and Asians plus other ethnicities. Cd concentration of raw leaf lettuce and iceberg lettuce were 0.066 and 0.051 mg/kg, respectively.	Average consumers: 1.7−5.3 μg/day. High consumers: 3.2−7.8 μg/day. Sources: Grains, beverages, vegetables, dairy, fruits, meat, and poultry plus fish contributed to 24.1%, 14.3%, 10.7%, 9.7%, 9.3% and 3.4% to total intake, respectively. High Pb foods: Chocolate syrup, liver, canned sweet potatoes, brownies, low-calorie buttermilk, salad dressing, raisins, English muffins, canned apricots, milk chocolate, candy bars, chocolate cake, chocolate chip cookies, wine and oat ring cereal with respective median levels of 14, 14, 14, 13, 13, 12, 10, 10, 9, 8, 8, 7 and 7 μg/kg.

A current tolerable Cd intake level established by FAO/WHO for the population of adults is 25 μg per kg body weight per month (58 μg per day for a 70-kg person) [41]. No tolerable Pb intake level has been identified after a previously established guideline was withdrawn in 2010 [41]. U.S. FDA interim safe intake level of Pb for the population of adults is 12.5 μg per day [42].

**Table 2:** Toxic exposure levels of cadmium and lead observed in various populations.

Countries	Exposure Levels and Estimates of Disease and Mortality Risks
U.S. [44,45,46,289]	[Cd]_u_ ≥ 1 µg/L, [Cd]_b_ ≥ 0.6 μg/L, [Pb]_b_ ≥ 2.4 μg/dL and [Cd]_b_ ≥ 0.6 plus [Pb]_b_ ≥ 2.4 μg/dL were associated with 1.48-, 1.32-,1.56- and 2.34-fold increment in CKD risk, respectively. [Cd]_b_ of >0.53 to >0.61 μg/L were associated with 1.80- to 2.2-fold increases in CKD risk.
U.S. [302,308,309,310,311,312,313,319,320,321]	E_Cd_ of ≥0.37 to ≥0.65 μg/g creatinine were associated with increased mortality from heart disease. E_Cd_ of >0.48 and ≥0.58 µg/g creatinine were linked to 4.29-fold and 3.22-fold increments in cancer mortality and lung cancer mortality in men, respectively. [Cd]_b_ > 0.6 μg/L was linked to a 3.83-fold increase in mortality from Alzheimer’s disease. [Pb]_b_ ≥ 5 μg/dL was linked to a 1.48-fold increment of cancer mortality[Pb]_b_ 1.0−6.7 μg/dL were linked to 1.37-, 1.70- and 2.08-fold increments of morality from all causes and cardiovascular and ischaemic heart diseases. [Pb]_u_ levels >1.26 μg/L were linked to 1.79- and 6.60-fold increments of mortality from all causes and cancer, respectively.
Sweden [291,292,314]	[Pb]_b_ ≥ 3.3 μg/dL was associated with a 1.49-fold rise of incidence of CKD and erythrocyte Pb was associated with developing ESKD. [Cd]_b_ ≥ 0.69 μg/L was linked to a 2.06-fold increase in mortality from all causes.
Australia [315]	A 2.7-fold higher [Cd]_u_ were linked to a 36% increase in mortality from heart failure and a 17% increase in the risk of having a heart failure event.
Japan [316,317,318,319]	E_Cd_ of ≥3.23 and ≥4.66 µg/g creatinine were linked to increased mortality by 64%, and 49% in men and women, respectively. The mortality from pancreatic cancer in women rose by 13% for every 1 μg/g creatinine increase in E_Cd_. In women with signs of Cd-related kidney pathologies, there were 3.85-, 7.71- and 10.1-fold increases in mortality from cancer of the uterus, kidney, and kidney plus urinary tract, respectively.
China [15,16]	Cd intake levels of 23.2, 29.6 and 36.9 μg/day were associated with 1.73-, 2.93- and 4.05-fold increments of CKD risk, For every 30 μg/day intake of Pb, all-cause mortality rose by 25%. Pb intake levels of 111.4 and 147 μg/day were linked to 1.52- and 3-fold increases in cancer mortality.
Thailand [53]	E_β2MG_ of 100–299, 300–999 and ≥1000 μg/g creatinine were associated with 4.66-, 6.16-, and 11.47-fold increases in CKD risk, compared with E_β2MG_ < 100 μg/g creatinine. An inverse association of E_β2_MG with eGFR was seen only in those with eGFR below 60 mL/min/1.73 m^2^, indicative of nephron loss. E_β2MG_ did not show an association with eGFR in those with normal eGFR.
Belgium [301]	Associations of [Cd]_u_ with [NAG]_u_ and [RBP]_u_ were seen in workers who had [Pb]_b_ ≥ 21.9 μg/dL, corresponding to the 75th percentile or higher.
Korea [300]	A correlation between [Cd]_b_ and [β_2_MG]_u_ was strengthened in those who had [Pb]_b_ above the median of 2.20 μg/dL.

[x]_u_ = urinary concentration of x; [x]_b_ = blood concentration of x; CKD = chronic kidney disease; ESKD = end stage kidney disease; E_Cd_ = excretion rate of Cd; β_2_MG = β_2_-microglobulin; E_β2MG_ = excretion rate of β_2_MG; NAG = N-acetyl-β-D-glucosaminidase. CKD is defined as estimated glomerular filtration rate < 60 mL/min/1.73 m^2^. E_β2MG_ ≥ 300 μg/g creatinine was the conventional cutoff value to define an adverse effect of excessive intake of Cd [41].

## Abbreviations

Ca	Calcium
Cd	Cadmium
Cu	Copper
Pb	Lead
Zn	Zn
MT	Metallothionein
PC	Phytochelatin
CdMT	Cadmium-metallothionein complex
CdPC	Cadmium-phytochelatin complex
GSH	Glutathione
δ-ALAD	Delta-aminolevulinic acid dehydratase
δ-ALA	Delta-aminolevulinic acid
AST	Aspartate aminotransferase
ALT	Alanine aminotransferase
β_2_MG	Beta_2_-microglobulin
KIM1	Kidney injury molecule 1
NAG	N-acetyl-β-D-glucosaminidase
RBP	Retinol-binding protein
GFR	Glomerular filtration rate, units of volume/time
eGFR	Estimated glomerular filtration rate, units of mL/min/1.73 m^2^
CKD-EPI	Chronic kidney disease epidemiology collaboration
JECFA	The Joint Expert Committee on Food Additives and Contaminants of the Food and Agriculture Organization and the World Health Organization of the United Nations
PTWI	Provisional tolerable weekly intake
TMI	Tolerable monthly intake
TDS	Total diet study
[x]_u_	Urinary concentration of x.
[x]_p_	Plasma concentration of x.
[x]_b_	Blood concentration of x.
[x]_k_	Kidney content of x.
C_cr_	Creatinine clearance, units of volume/time
V_u_	Urine flow rate, units of volume/time
E*_x_*/C_cr_	Excretion rate of *x* per volume of filtrate, units of mass/volume, where *x* = Cd, NAG, or β_2_MG
FE_β2MG_	Fractional excretion of β_2_MG, %
FR_β2MG_	Fractional reabsorption of β_2_MG, %

## References

[1] Satarug S., Vesey D.A., Gobe G.C. (2017). Health risk assessment of dietary cadmium intake: Do current guidelines indicate how much is safe?. Environ. Health Perspect..

[2] Satarug S., Vesey D.A., Gobe G.C. (2017). Current health risk assessment practice for dietary cadmium: Data from different countries. Food Chem. Toxicol..

[3] Shefa S.T., Héroux P. (2017). Both physiology and epidemiology support zero tolerable blood lead levels. Toxicol. Lett..

[4] Daley G.M., Pretorius C.J., Ungerer J.P. (2018). Lead toxicity: An Australian perspective. Clin. Biochem. Rev..

[5] World Health Organization (WHO) Preventing Disease through Healthy Environments: Ten Chemicals of Major Public Health Concern; Public Environment WHO: Geneva, Switzerland. https://www.who.int/ipcs/features/10chemicals_en.pdf?ua=1.

[6] Satarug S., Haswell-Elkins M.R., Moore M.R. (2000). Safe levels of cadmium intake to prevent renal toxicity in human subjects. Br. J. Nutr..

[7] Satarug S., Baker J.R., Urbenjapol S., Haswell-Elkins M., Reilly P.E., Williams D.J., Moore M.R. (2003). A global perspective on cadmium pollution and toxicity in non-occupationally exposed population. Toxicol. Lett..

[8] Satarug S. (2018). Dietary cadmium intake and its effects on kidneys. Toxics.

[9] Satarug S. (2012). Long-term exposure to cadmium in food and cigarette smoke, liver effects and hepatocellular carcinoma. Curr. Drug Metab..

[10] Satarug S., Moore M.R. (2012). Emerging roles of cadmium and heme oxygenase in type-2 diabetes and cancer susceptibility. Tohoku J. Exp. Med..

[11] Gibb H.J., Barchowsky A., Bellinger D., Bolger P.M., Carrington C., Havelaar A.H., Oberoi S., Zang Y., O’Leary K., Devleesschauwer B. (2019). Estimates of the 2015 global and regional disease burden from four foodborne metals-arsenic, cadmium, lead and methylmercury. Environ. Res..

[12] Satarug S., Gobe G.C., Ujjin P., Vesey D.A. (2020). A comparison of the nephrotoxicity of low doses of cadmium and lead. Toxics.

[13] Wang X., Ding N., Tucker K.L., Weisskopf M.G., Sparrow D., Hu H., Park S.K. (2017). A Western diet pattern is associated with higher concentrations of blood and bone lead among middle-aged and elderly men. J. Nutr..

[14] Ding N., Wang X., Tucker K.L., Weisskopf M.G., Sparrow D., Hu H., Park S.K. (2019). Dietary patterns, bone lead and incident coronary heart disease among middle-aged to elderly men. Environ. Res..

[15] Shi Z., Taylor A.W., Riley. M., Byles. J., Liu J., Noakes M. (2018). Association between dietary patterns, cadmium intake and chronic kidney disease among adults. Clin. Nutr..

[16] Shi Z., Zhen S., Orsini N., Zhou Y., Zhou Y., Liu J., Taylor A.W. (2017). Association between dietary lead intake and 10-year mortality among Chinese adults. Environ. Sci. Pollut. Res..

[17] Gobe G., Crane D. (2010). Mitochondria, reactive oxygen species and cadmium toxicity in the kidney. Toxicol. Lett..

[18] Nair A.R., Lee W.K., Smeets K., Swennen Q., Sanchez A., Thévenod F., Cuypers A. (2015). Glutathione and mitochondria determine acute defense responses and adaptive processes in cadmium-induced oxidative stress and toxicity of the kidney. Arch. Toxicol..

[19] Matović V., Buha A., Ðukić-Ćosić D., Bulat Z. (2015). Insight into the oxidative stress induced by lead and/or cadmium in blood, liver and kidneys. Food Chem. Toxicol..

[20] Satarug S., Vesey D.A., Gobe G.C. (2017). Kidney cadmium toxicity, diabetes and high blood pressure: The perfect storm. Tohoku J. Exp. Med..

[21] Garza-Lombó C., Posadas Y., Quintanar L., Gonsebatt M.E., Franco R. (2018). Neurotoxicity linked to dysfunctional metal ion homeostasis and xenobiotic metal exposure: Redox signaling and oxidative stress. Antioxid. Redox Signal..

[22] Valko M., Jomova K., Rhodes C.J., Kuča K., Musílek K. (2016). Redox- and non-redox-metal-induced formation of free radicals and their role in human disease. Arch. Toxicol..

[23] Moulis J.M., Bourguinon J., Catty P., Wolfgang M., Anthony W. (2014). Chapter 23 Cadmium. RSC Metallobiology Series No. 2, Binding, Transport. and Storage of Metal. Ions in Biological Cells.

[24] Cangelosi V., Pecoraro V., Wolfgang M., Anthony W. (2014). Chapter 28 Lead. RSC Metallobiology Series No. 2, Binding, Transport. and Storage of Metal. Ions in Biological Cells.

[25] Sanders T., Liu Y., Buchner V., Tchounwou P.B. (2009). Neurotoxic effects and biomarkers of lead exposure: A review. Rev. Environ. Health.

[26] Carpenter M.C., Shami Shah A., DeSilva S., Gleaton A., Su A., Goundie B., Croteau M.L., Stevenson M.J., Wilcox D.E., Austin R.N. (2016). Thermodynamics of Pb(ii) and Zn(ii) binding to MT-3, a neurologically important metallothionein. Metallomics.

[27] Satarug S., Baker J.R., Reilly P.E., Esumi H., Moore M.R. (2000). Evidence for a synergistic interaction between cadmium and endotoxin toxicity and for nitric oxide and cadmium displacement of metals in the kidney. Nitric Oxide.

[28] Satarug S., Baker J.R., Reilly P.E., Moore M.R., Williams D.J. (2001). Changes in zinc and copper homeostasis in human livers and kidneys associated with exposure to environmental cadmium. Hum. Exp. Toxicol..

[29] Satarug S., Nishijo M., Ujjin P., Moore M.R. (2018). Chronic exposure to low-level cadmium induced zinc-copper dysregulation. J. Trace Elem. Med. Biol..

[30] Prozialeck W.C., Lamar P.C., Edwards J.R. (2016). Effects of sub-chronic Cd exposure on levels of copper, selenium, zinc, iron and other essential metals in rat renal cortex. Toxicol. Rep..

[31] Thevenod F. (2003). Nephrotoxicity and the proximal tubule. Insights from cadmium. Nephron Physiol..

[32] Moulis J.M. (2010). Cellular mechanisms of cadmium toxicity related to the homeostasis of essential metals. Biometals.

[33] Nzengue Y., Candéias S.M., Sauvaigo S., Douki T., Favier A., Rachidi W., Guiraud P. (2011). The toxicity redox mechanisms of cadmium alone or together with copper and zinc homeostasis alteration: Its redox biomarkers. J. Trace Elem. Med. Biol..

[34] Nzengue Y., Steiman R., Rachidi W., Favier A., Guiraud P. (2012). Oxidative stress induced by cadmium in the C6 cell line: Role of copper and zinc. Biol. Trace Elem. Res..

[35] Eom S.Y., Yim D.H., Huang M., Park C.H., Kim G.B., Yu S.D., Choi B.S., Park J.D., Kim Y.D., Kim H. (2020). Copper-zinc imbalance induces kidney tubule damage and oxidative stress in a population exposed to chronic environmental cadmium. Int. Arch. Occup. Environ. Health.

[36] Rubino F.M. (2015). Toxicity of glutathione-binding metals: A review of targets and mechanisms. Toxics.

[37] Phillips J.D. (2019). Heme biosynthesis and the porphyrias. Mol. Genet. Metab..

[38] Tobwala S., Wang H.-J., Carey J.W., Banks W.A., Ercal N. (2014). Effects of lead and cadmium on brain endothelial cell survival, monolayer permeability, and crucial oxidative stress markers in an in vitro model of the blood-brain barrier. Toxics.

[39] Wang W., Duan B., Xu H., Xu L., Xu T.L. (2006). Calcium-permeable acid-sensing ion channel is a molecular target of the neurotoxic metal ion lead. J. Biol. Chem..

[40] FAO/WHO (1993). Evaluation of Certain Food Additives and Contaminants (Forty-First Report of the Joint FAO/WHO Expert Committee on Food Additives).

[41] Food and Agriculture Organization of the United Nations (FAO), World Health Organization (WHO) Summary and Conclusions. Proceedings of the Joint FAO/WHO Expert Committee on Food Additives Seventy-Third Meeting.

[42] Flannery B.M., Dolan L.C., Hoffman-Pennesi D., Gavelek A., Jones O.E., Kanwal R., Wolpert B., Gensheimer K., Dennis S., Fitzpatrick S.U.S. (2020). Food and Drug Administration’s interim reference levels for dietary lead exposure in children and women of childbearing age. Regul. Toxicol. Pharmacol..

[43] Dolan L.C., Flannery B.M., Hoffman-Pennesi D., Gavelek A., Jones O.E., Kanwal R., Wolpert B., Gensheimer K., Dennis S., Fitzpatrick S. (2020). A review of the evidence to support interim reference level for dietary lead exposure in adults. Regul. Toxicol. Pharmacol..

[44] Ferraro P.M., Costanzi S., Naticchia A., Sturniolo A., Gambaro G. (2010). Low level exposure to cadmium increases the risk of chronic kidney disease: Analysis of the NHANES 1999–2006. BMC Public Health.

[45] Lin Y.S., Ho W.C., Caffrey J.L., Sonawane B. (2014). Low serum zinc is associated with elevated risk of cadmium nephrotoxicity. Environ. Res..

[46] Madrigal J.M., Ricardo A.C., Persky V., Turyk M. (2018). Associations between blood cadmium concentration and kidney function in the U.S. population: Impact of sex, diabetes and hypertension. Environ. Res..

[47] Crinnion W.J. (2010). The CDC fourth national report on human exposure to environmental chemicals: What it tells us about our toxic burden and how it assists environmental medicine physicians. Altern. Med. Rev..

[48] Levey A.S., Stevens L.A., Schmid C.H., Zhang Y., Castro A.F., Feldman H.I., Kusek J.W., Eggers P., Van Lente F., Greene T. (2009). A new equation to estimate glomerular filtration rate. Ann. Intern. Med..

[49] Levey A.S., Inker L.A., Coresh J. (2014). GFR estimation: From physiology to public health. Am. J. Kidney Dis..

[50] Levey A.S., Becker C., Inker L.A. (2015). Glomerular filtration rate and albuminuria for detection and staging of acute and chronic kidney disease in adults: A systematic review. JAMA.

[51] Satarug S., Ruangyuttikarn W., Nishijo M., Ruiz P. (2018). Urinary cadmium threshold to prevent kidney disease development. Toxics.

[52] Satarug S., Boonprasert K., Gobe G.C., Ruenweerayut R., Johnson D.W., Na-Bangchang K., Vesey D.A. (2018). Chronic exposure to cadmium is associated with a marked reduction in glomerular filtration rate. Clin. Kidney J..

[53] Satarug S., Vesey D.A., Nishijo M., Ruangyuttikarnm W., Gobe G.C. (2019). The inverse association of glomerular function and urinary β_2_-MG excretion and its implications for cadmium health risk assessment. Environ. Res..

[54] Satarug S., Vesey D.A., Ruangyuttikarn W., Nishijo M., Gobe G.C., Phelps K.R. (2019). The source and pathophysiologic significance of excreted cadmium. Toxics.

[55] Järup L. (2003). Hazards of heavy metal contamination. Br. Med. Bull..

[56] Wu S., Deng F., Hao Y., Shima M., Wang X., Zheng C., Wei H., Lv H., Lu X., Huang J. (2013). Chemical constituents of fine particulate air pollution and pulmonary function in healthy adults: The Healthy Volunteer Natural Relocation study. J. Hazard. Mater..

[57] Jung M.S., Kim J.Y., Lee H.S., Lee C.G., Song H.S. (2016). Air pollution and urinary N-acetyl-β-glucosaminidase levels in residents living near a cement plant. Ann. Occup. Environ. Med..

[58] Jin Y., Lu Y., Li Y., Zhao H., Wang X., Shen Y., Kuang X. (2020). Correlation between environmental low-dose cadmium exposure and early kidney damage: A comparative study in an industrial zone vs. a living quarter in Shanghai, China. Environ.Toxicol. Pharmacol..

[59] Repić A., Bulat P., Antonijević B., Antunović M., Džudović J., Buha A., Bulat Z. (2020). The influence of smoking habits on cadmium and lead blood levels in the Serbian adult people. Environ. Sci. Pollut. Res. Int..

[60] Dumkova J., Vrlikova L., Vecera Z., Putnova B., Docekal B., Mikuska P., Fictum P., Hampl A., Buchtova M. (2016). Inhaled cadmium oxide nanoparticles: Their in vivo fate and effect on target organs. Int. J. Mol. Sci..

[61] Dumková J., Smutná T., Vrlíková L., Le Coustumer P., Večeřa Z., Dočekal B., Mikuška P., Čapka L., Fictum P., Hampl A. (2017). Sub-chronic inhalation of lead oxide nanoparticles revealed their broad distribution and tissue-specific subcellular localization in target organs. Part. Fibre Toxicol..

[62] Tulinska J., Masanova V., Liskova A., Mikusova M.L., Rollerova E., Krivosikova Z., Stefikova K., Uhnakova I., Ursinyova M., Babickova J. (2020). Six-week inhalation of CdO nanoparticles in mice: The effects on immune response, oxidative stress, antioxidative defense, fibrotic response, and bones. Food Chem. Toxicol..

[63] Sutunkova M.P., Solovyeva S.N., Chernyshov I.N., Klinova S.V., Gurvich V.B., Shur V.Y., Shishkina E.V., Zubarev I.V., Privalova L.I., Katsnelson B.A. (2020). Manifestation of systemic toxicity in rats after a short-time inhalation of lead oxide nanoparticles. Int. J. Mol. Sci..

[64] Zahran S., McElmurry S.P., Sadler R.C. (2017). Four phases of the Flint qater crisis: Evidence from blood lead levels in children. Environ. Res..

[65] Roy S., Tang M., Edwards M.A. (2019). Lead release to potable water during the Flint, Michigan water crisis as revealed by routine biosolids monitoring data. Water Res..

[66] Bandara J.M., Wijewardena H.V., Liyanege J., Upul M.A., Bandara J.M. (2010). Chronic renal failure in Sri Lanka caused by elevated dietary cadmium: Trojan horse of the green revolution. Toxicol. Lett..

[67] Kader M., Lamb D.T., Mahbub K.R., Megharaj M., Naidu R. (2016). Predicting plant uptake and toxicity of lead (Pb) in long-term contaminated soils from derived transfer functions. Environ. Sci. Pollut. Res. Int..

[68] Lamb D.T., Kader M., Ming H., Wang L., Abbasi S., Megharaj M., Naidu R. (2016). Predicting plant uptake of cadmium: Validated with long-term contaminated soils. Ecotoxicology.

[69] Wilkinson J.M., Hill J., Phillips C.J. (2003). The accumulation of potentially-toxic metals by grazing ruminants. Proc. Nutr. Soc..

[70] Bischoff K., Hillebrandt J., Erb H.N., Thompson B., Johns S. (2016). Comparison of blood and tissue lead concentrations from cattle with known lead exposure. Food Addit. Contam. Part A Chem. Anal. Control. Expo. Risk Assess..

[71] Centers for Disease Control and Prevention (2012). CDC Response to Advisory Committee on Childhood Lead Poisoning Prevention Recommendations in “Low Level Lead Exposure Harms Children: A Renewed Call of Primary Prevention”. http://www.cdc.gov/nceh/lead/ACCLPP/CDC_Response_Lead_Exposure_Recs.pdf.

[72] Feng C.X., Cao. J., Bendell L. (2011). Exploring spatial and temporal variations of cadmium concentrations in pacific oysters from British Columbia. Biometrics.

[73] Losasso C., Bille L., Patuzzi I., Lorenzetto M., Binato G., Pozza M.D., Ferrè N., Ricci N. (2015). Possible influence of natural events on heavy metals exposure from shellfish consumption: A case study in the north-east of Italy. Front. Public Health.

[74] Guéguen M., Amiard J.-C., Arnich N., Badot P.-M., Claisse D., Guérin T., Vernoux J.-P. (2011). Shellfish and residual chemical contaminants: Hazards, monitoring, and health risk assessment along French coasts. Rev. Environ. Contam. Toxicol..

[75] Burioli E.A.V., Squadrone S., Stella C., Foglini C., Abete M.C., Prearo M. (2017). Trace element occurrence in the Pacific oyster Crassostrea gigas from coastal marine ecosystems in Italy. Chemosphere.

[76] Renieri E.A., Alegakis A.K., Kiriakakis M., Vinceti M., Ozcagli E., Wilks M.F., Tsatsakis A.M. (2014). Cd, Pb and Hg biomonitoring in fish of the Mediterranean region and risk estimations on fish consumption. Toxics.

[77] Cobbett C.S. (2000). Phytochelatins and their roles in heavy metal detoxification. Plant. Physiol..

[78] Cobbett C., Goldsbrough P. (2002). Phytochelatins and metallothioneins: Roles in heavy metal detoxification and homeostasis. Annu. Rev. Plant Biol..

[79] Pivato M., Fabrega-Prats M., Masi A. (2014). Low-molecular-weight thiols in plants: Functional and analytical implications. Arch. Biochem. Biophys..

[80] Klaassen C.D., Liu J., Diwan B.A. (2009). Metallothionein protection of cadmium toxicity. Toxicol. Appl. Pharmacol..

[81] Scott S.R., Smith K.E., Dahman C., Gorski P.R., Adams S.V., Shafer M.M. (2019). Cd isotope fractionation during tobacco combustion produces isotopic variation outside the range measured in dietary sources. Sci. Total Environ..

[82] Aoshima K. (1987). Epidemiology and tubular dysfunction in the inhabitants of a cadmium-polluted area in the Jinzu River basin in Toyama Prefecture. Tohoku J. Exp. Med..

[83] Spungen J.H. (2019). Children’s exposures to lead and cadmium: FDA total diet study 2014–2016. Food Addit. Contam. Part A Chem. Anal. Control. Expo. Risk Assess..

[84] Gavelek A., Spungen J., Hoffman-Pennesi D., Flannery B., Dolan L., Dennis S., Fitzpatrick S. (2020). Lead exposures in older children (males and females 7–17 years), women of childbearing age (females 16–49 years) and adults (males and females 18+ years): FDA total diet study 2014-16. Food Addit. Contam. Part A Chem. Anal. Control. Expo. Risk Assess..

[85] European Food Safety Agency (EFSA) (2011). Statement on tolerable weekly intake for cadmium. EFSA J..

[86] European Food Safety Agency (EFSA) (2012). Cadmium dietary exposure in the European population. EFSA J..

[87] Callan A., Hinwood A., Devine A. (2014). Metals in commonly eaten groceries in Western Australia: A market basket survey and dietary assessment. Food Addit. Contam. Part A Chem. Anal. Control. Expo. Risk Assess..

[88] Sand S., Becker W. (2012). Assessment of dietary cadmium exposure in Sweden and population health concern including scenario analysis. Food Chem. Toxicol..

[89] Wei J., Gao J., Cen K. (2019). Levels of eight heavy metals and health risk assessment considering food consumption by China’s residents based on the 5th China total diet study. Sci. Total Environ..

[90] Xiao G., Liu Y., Dong K.F., Lu J. (2020). Regional characteristics of cadmium intake in adult residents from the 4th and 5th Chinese total diet study. Environ. Sci. Pollut. Res. Int..

[91] Jin Y., Liu P., Sun J., Wang C., Min J., Zhang Y., Wang S., Wu Y. (2014). Dietary exposure and risk assessment to lead of the population of Jiangsu province, China. Food Addit. Contam. Part A Chem. Anal. Control. Expo. Risk Assess..

[92] Lim J.A., Kwon H.J., Ha M., Kim H., Oh S.Y., Kim J.S., Lee S.A., Park J.D., Hong Y.S., Sohn S.J. (2015). Korean research project on the integrated exposure assessment of hazardous substances for food safety. Environ. Health Toxicol..

[93] Kim H., Lee J., Woo H.D., Kim D.W., Choi I.J., Kim Y.I., Kim J. (2019). Association between dietary cadmium intake and early gastric cancer risk in a Korean population: A case-control study. Eur. J. Nutr..

[94] Schwarz M.A., Lindtner O., Blume K., Heinemeyer G., Schneider K. (2014). Cadmium exposure from food: The German LExUKon project. Food Addit. Contam. Part A Chem. Anal. Control. Expo. Risk Assess..

[95] Marín S., Pardo O., Báguena R., Font G., Yusà V. (2017). Dietary exposure to trace elements and health risk assessment in the region of Valencia, Spain: A total diet study. Food Addit. Contam. Part A Chem. Anal. Control. Expo. Risk Assess..

[96] Puerto-Parejo L.M., Aliaga I., Canal-Macias M.L., Leal-Hernandez O., Roncero-Martín R., Rico-Martín S., Moran J.M. (2017). Evaluation of the dietary intake of cadmium, lead and mercury and its relationship with bone health among postmenopausal women in Spain. Int. J. Environ. Res. Public Health.

[97] Kim K., Melough M.M., Vance T.M., Noh H., Koo S.I., Chun O.K. (2018). Dietary cadmium intake and sources in the US. Nutrients.

[98] Adams S.V., Quraishi S.M., Shafer M.M., Passarelli M.N., Freney E.P., Chlebowski R.T., Luo J., Meliker J.R., Mu L., Neuhouser M.L. (2014). Dietary cadmium exposure and risk of breast, endometrial, and ovarian cancer in the Women’s Health Initiative. Environ. Health Perspect..

[99] Filippini T., Cilloni S., Malavolti M., Violi F., Malagoli C., Tesauro M., Bottecchi I., Ferrari A., Vescovi L., Vinceti M. (2018). Dietary intake of cadmium, chromium, copper, manganese, selenium and zinc in a Northern Italy community. J. Trace Elem. Med. Biol..

[100] Schneider K., Schwarz M.A., Lindtner O., Blume K., Heinemeyer G. (2014). Lead exposure from food: The German LExUKon. Food Addit. Contam. Part A Chem. Anal. Control. Expo. Risk Assess..

[101] Arnich N., Sirot V., Rivière G., Jean J., Noël L., Guérin T., Leblanc J.-C. (2012). Dietary exposure to trace elements and health risk assessment in the 2nd French Total Diet Study. Food Chem. Toxicol..

[102] Vromman V., Waegeneers N., Cornelis C., De Boosere I., Van Holderbeke M., Vinkx C., Smolders E., Huyghebaert A., Pussemier L. (2010). Dietary cadmium intake by the Belgian adult population. Food Addit. Contam. Part A Chem. Anal. Control. Expo. Risk Assess..

[103] Horiguchi H., Oguma E., Sasaki S., Miyamoto K., Hosoi Y., Ono A., Kayama F. (2020). Exposure assessment of cadmium in female farmers in cadmium-polluted areas in Northern Japan. Toxics.

[104] Nishito Y., Kambe T. (2018). Absorption mechanisms of iron, copper, and zinc: An overview. J. Nutr. Sci. Vitaminol..

[105] Vesey D.A. (2010). Transport pathways for cadmium in the intestine and kidney proximal tubule: Focus on the interaction with essential metals. Toxicol. Lett..

[106] Thévenod F., Lee W.-K., Garrick M.D. (2020). Iron and cadmium entry into renal mitochondria: Physiological and toxicological implications. Front. Cell Develop. Biol..

[107] Kovacs G., Danko T., Bergeron M.J., Balazs B., Suzuki Y., Zsembery A., Hediger M.A. (2011). Heavy metal cations permeate the TRPV6 epithelial cation channel. Cell Calcium..

[108] Kovacs G., Montalbetti N., Franz M.C., Graeter S., Simonin A., Hediger M.A. (2013). Human TRPV5 and TRPV6: Key players in cadmium and zinc toxicity. Cell Calcium..

[109] Fujishiro H., Hamao S., Tanaka R., Kambe T., Himeno S. (2017). Concentration-dependent roles of DMT1 and ZIP14 in cadmium absorption in Caco-2 cells. J. Toxicol. Sci..

[110] Thevenod F., Fels J., Lee W.-K., Zarbock R. (2019). Channels, transporters and receptors for cadmium and cadmium complexes in eukaryotic cells: Myths and facts. Biometals.

[111] Mackenzie B., Takanaga H., Hubert N., Rolfs A., Hediger M.A. (2007). Functional properties of multiple isoforms of human divalent metal-ion transporter 1 (DMT1). Biochem. J..

[112] Illing A.C., Shawki A., Cunningham C.L., Mackenzie B. (2012). Substrate profile and metal-ion selectivity of human divalent metal-ion transporter-1. J. Biol. Chem..

[113] Bannon D.I., Abounader R., Lees P.S., Bressler J.P. (2003). Effect of DMT1 knockdown on iron, cadmium, and lead uptake in Caco-2 cells. Am. J. Physiol. Cell Physiol..

[114] Aduayom I., Jumarie C. (2005). Reciprocal inhibition of Cd and Pb sulfocomplexes for uptake in Caco-2 cells. J. Biochem. Mol. Toxicol..

[115] Mitchell C.J., Shawki A., Ganz T., Nemeth E., Mackenzie B. (2014). Functional properties of human ferroportin, a cellular iron exporter reactive also with cobalt and zinc. Am. J. Physiol. Cell Physiol..

[116] Jeon H.-K., Jin H.-S., Lee D.-H., Choi W.-S., Moon C.-K., Oh Y.J., Lee T.H. (2004). Proteome analysis associated with cadmium adaptation in U937 cells: Identification of calbindin-D28k as a secondary cadmium-responsive protein that confers resistance to cadmium-induced apoptosis. J. Biol. Chem..

[117] Fujita Y., ElBelbasi H.I., Min K.-S., Onosaka S., Okada Y., Matsumoto Y., Mutoh N., Tanaka K. (1993). Fate of cadmium bound to phytochelatin in rats. Res. Commun. Chem. Pathol. Pharmacol..

[118] Langelueddecke C., Roussa E., Fenton R.A., Thévenod F. (2013). Expression and function of the lipocalin-2 (24p3/NGAL) receptor in rodent and human intestinal epithelia. PLoS ONE.

[119] Langelueddecke C., Lee W.-K., Thevenod F. (2014). Differential transcytosis and toxicity of the hNGAL receptor ligands cadmium-metallothionein and cadmium-phytochelatin in colon-like Caco-2 cells: Implications for cadmium toxicity. Toxicol. Lett..

[120] Jorge-Nebert L.F., Gálvez-Peralta M., Figueroa J.L., Somarathna M., Hojyo S., Fukada T., Nebert D.W. (2015). Comparing gene expression during cadmium uptake and distribution: Untreated versus oral Cd-treated wild-type and ZIP14 knockout mice. Toxicol. Sci..

[121] McKenna I.M., Gordon T., Chen L.C., Anver M.R., Waalkes M.P. (1998). Expression of metallothionein protein in the lungs of Wistar rats and C57 and DBA mice exposed to cadmium oxide fumes. Toxicol. Appl. Pharmacol..

[122] Takeda K., Fujita H., Shibahara S. (1995). Differential control of the metal-mediated activation of the human heme oxygenase-1 and metallothionein IIA genes. Biochem. Biophys. Res. Commun..

[123] Hart B.A. (1986). Cellular and biochemical response of the rat lung to repeated inhalation of cadmium. Toxicol. Appl. Pharmacol..

[124] Hart B.A., Gong Q., Eneman J.D. (1996). Pulmonary metallothionein expression in rats following single and repeated exposure to cadmium aerosols. Toxicology.

[125] Chandler J.D., Wongtrakool C., Banton S.A., Li S., Orr M.L., Barr D.B., Neujahr D.C., Sutliff R.L., Go Y.M., Jones D.P. (2016). Low-dose oral cadmium increases airway reactivity and lung neuronal gene expression in mice. Physiol. Rep..

[126] Sabolić I., Breljak D., Skarica M., Herak-Kramberger C.M. (2010). Role of metallothionein in cadmium traffic and toxicity in kidneys and other mammalian organs. Biometals.

[127] Yu J., Fujishiro H., Miyataka H., Oyama T.M., Hasegawa T., Seko Y., Miura N., Himeno S. (2009). Dichotomous effects of lead acetate on the expression of metallothionein in the liver and kidney of mice. Biol. Pharm. Bull..

[128] Dai S., Yin Z., Yuan G., Lu H., Jia R., Xu J., Song X., Li L., Shu Y., Liang X. (2013). Quantification of metallothionein on the liver and kidney of rats by subchronic lead and cadmium in combination. Environ. Toxicol. Pharmacol..

[129] Kikuchi Y., Nomiyama T., Kumagai N., Dekio F., Uemura T., Takebayashi T., Nishiwaki Y., Matsumoto Y., Sano Y., Hosoda K. (2003). Uptake of cadmium in meals from the digestive tract of young non-smoking Japanese female volunteers. J. Occup. Health.

[130] Wang X., Kim D., Tucker K.L., Weisskopf M.G., Sparrow D., Hu H., Park S.K. (2019). Effect of dietary sodium and potassium on the mobilization of bone lead among middle-aged and older men: The Veterans Affairs Normative Aging Study. Nutrients.

[131] Nielsen R., Christensen E.I., Birn H. (2016). Megalin and cubilin in proximal tubule protein reabsorption: From experimental models to human disease. Kidney Int..

[132] Onodera A., Tani M., Michigami T., Yamagata M., Min K.S., Tanaka K., Nakanishi T., Kimura T., Itoh N. (2012). Role of megalin and the soluble form of its ligand RAP in Cd-metallothionein endocytosis and Cd-metallothionein-induced nephrotoxicity in vivo. Toxicol. Lett..

[133] Langelueddecke C., Roussa E., Fenton R.A., Wolff N.A., Lee W.K., Thévenod F. (2012). Lipocalin-2 (24p3/neutrophil gelatinase-associated lipocalin (NGAL)) receptor is expressed in distal nephron and mediates protein endocytosis. J. Biol. Chem..

[134] Fels J., Scharner B., Zarbock R., Zavala Guevara I.P., Lee W.K., Barbier O.C., Thévenod F. (2019). Cadmium complexed with β2-microglubulin, albumin and lipocalin-2 rather than metallothionein cause megalin:cubilin dependent toxicity of the renal proximal tubule. Int. J. Mol. Sci..

[135] Nascimento C.R.B., Risso W.E., Martinez C.B.D.R. (2016). Lead accumulation and metallothionein content in female rats of different ages and generations after daily intake of Pb-contaminated food. Environ. Toxicol. Pharmacol..

[136] Satarug S., Baker J.R., Reilly P.E., Moore M.R., Williams D.J. (2002). Cadmium levels in the lung, liver, kidney cortex, and urine samples from Australians without occupational exposure to metals. Arch. Environ. Health.

[137] Baker J.R., Edwards R.J., Lasker J.M., Moore M.R., Satarug S. (2005). Renal and hepatic accumulation of cadmium and lead in the expression of CYP4F2 and CYP2E1. Toxicol. Lett..

[138] Barregard L., Fabricius-Lagging E., Lundh T., Mölne J., Wallin M., Olausson M., Modigh C., Sallstenm G. (2010). Cadmium, mercury, and lead in kidney cortex of living kidney donors: Impact of different exposure sources. Environ. Res..

[139] Järup L., Rogenfelt A., Elinder C.G., Nogawa K., Kjellström T. (1983). Biological half-time of cadmium in the blood of workers after cessation of exposure. Scand. J. Work Environ. Health.

[140] Börjesson J., Bellander T., Järup L., Elinder C.G., Mattsson S. (1997). In vivo analysis of cadmium in battery workers versus measurements of blood, urine, and workplace air. Occup. Environ. Med..

[141] Suwazono Y., Kido T., Nakagawa H., Nishijo M., Honda R., Kobayashi E., Dochi M., Nogawa K. (2009). Biological half-life of cadmium in the urine of inhabitants after cessation of cadmium exposure. Biomarkers.

[142] Ishizaki M., Suwazono Y., Kido T., Nishijo M., Honda R., Kobayashi E., Nogawa K., Nakagawa H. (2015). Estimation of biological half-life of urinary cadmium in inhabitants after cessation of environmental cadmium pollution using a mixed linear model. Food Addit. Contam. Part A Chem. Anal. Control. Expo. Risk Assess..

[143] Fransson M.N., Barregard L., Sallsten G., Akerstrom M., Johanson G. (2014). Physiologically-based toxicokinetic model for cadmium using Markov-chain Monte Carlo analysis of concentrations in blood, urine, and kidney cortex from living kidney donors. Toxicol. Sci..

[144] Specht A.J., Lin Y., Weisskopf M., Yan C., Hu H., Xu J., Nie L.H. (2016). XRF-measured bone lead (Pb) as a biomarker for Pb exposure and toxicity among children diagnosed with Pb poisoning. Biomarkers.

[145] Orlowski C., Piotrowski J.K., Subdys J.K., Gross A. (1998). Urinary cadmium as indicator of renal cadmium in humans: An autopsy study. Hum. Exp. Toxicol..

[146] Akerstrom M., Barregard L., Lundh T., Sallsten G. (2013). The relationship between cadmium in kidney and cadmium in urine and blood in an environmentally exposed population. Toxicol. Appl. Pharmacol..

[147] Wallin M., Sallsten G., Lundh T., Barregard L. (2014). Low-level cadmium exposure and effects on kidney function. Occup. Environ. Med..

[148] Gerhardsson L., Englyst V., Lundström N.G., Sandberg S., Nordberg G. (2002). Cadmium, copper and zinc in tissues of deceased copper smelter workers. J. Trace Elem. Med. Biol..

[149] Lou M., Garay R., Alda. J.O. (1991). Cadmium uptake through the anion exchanger in human red blood cells. J. Physiol..

[150] Wu F., Satchwell T.J., Toye A.M. (2011). Anion exchanger 1 in red blood cells and kidney: Band 3’s in a pod. Biochem. Cell Biol..

[151] Parker M.D., Boron W.F. (2013). The divergence, actions, roles, and relatives of sodium-coupled bicarbonate transporters. Physiol. Rev..

[152] Savigni D.L., Morgan E.H. (1998). Transport mechanisms for iron and other transition metals in rat and rabbit erythroid cells. J. Physiol..

[153] Simons T.J. (1986). The role of anion transport in the passive movement of lead across the human red cell membrane. J. Physiol..

[154] Simons T.J. (1993). Lead transport and binding by human erythrocytes in vitro. Pflugers Arch..

[155] Lang F., Abed M., Lang E., Föller M. (2014). Oxidative stress and suicidal erythrocyte death. Antioxid. Redox Signal..

[156] Lang E., Lang F. (2015). Mechanisms and pathophysiological significance of eryptosis, the suicidal erythrocyte death. Semin. Cell Dev. Biol..

[157] Attanzio A., Frazzitta A., Vasto S., Tesoriere L., Pintaudi A.M., Livrea M.A., Cilla A., Allegra M. (2019). Increased eryptosis in smokers is associated with the antioxidant status and C-reactive protein levels. Toxicology.

[158] Scott B.J., Bradwell A.R. (1983). Identification of the serum binding proteins for iron, zinc, cadmium, nickel, and calcium. Clin. Chem..

[159] Horn N.M., Thomas A.L. (1996). Interactions between the histidine stimulation of cadmium and zinc influx into human erythrocytes. J. Physiol..

[160] Turell L., Radi R., Alvarez B. (2013). The thiol pool in human plasma: The central contribution of albumin to redox processes. Free Radic. Biol. Med..

[161] Morris T.T., Keir J.L., Boshart S.J., Lobanov V.P., Ruhland A.M., Bahl N., Gailer J. (2014). Mobilization of Cd from human serum albumin by small molecular weight thiols. J. Chromatogr. B Anal. Technol. Biomed. Life Sci..

[162] Sagmeister P., Gibson M.A., McDade K.H., Gailer J. (2016). Physiologically relevant plasma d,l-homocysteine concentrations mobilize Cd from human serum albumin. J. Chromatogr. B Anal. Technol. Biomed. Life Sci..

[163] Gaudet M.M., Deubler E.L., Kelly R.S., Diver W.R., Teras L.R., Hodge J.M., Levine K.E., Haines L.G., Lundh T., Lenner P. (2019). Blood levels of cadmium and lead in relation to breast cancer risk in three prospective cohorts. Int. J. Cancer.

[164] Lin J., Zhang F., Lei Y. (2016). Dietary intake and urinary level of cadmium and breast cancer risk: A meta-analysis. Cancer Epidemiol..

[165] Rokadia H.K., Agarwal S. (2013). Serum heavy metals and obstructive lung disease: Results from the National Health and Nutrition Examination Survey. Chest.

[166] Yang G., Sun T., Han Y.Y., Rosser F., Forno E., Chen W., Celedón J.C. (2019). Serum cadmium and lead, current wheeze, and lung function in a nationwide study of adults in the United States. J. Allergy Clin. Immunol. Pract..

[167] Bergdahl I.A., Schütz A., Gerhardsson L., Jensen A., Skerfving S. (1997). Lead concentrations in human plasma, urine and whole blood. Scand. J. Work Environ. Health.

[168] Manton W.I., Rothenberg S.J., Manalo M. (2001). The lead content of blood serum. Environ. Res..

[169] Smith D., Hernandez-Avila M., Téllez-Rojo M.M., Mercado A., Hu H. (2002). The relationship between lead in plasma and whole blood in women. Environ. Health Perspect..

[170] Barbosa F., Tanus-Santos J.E., Gerlach R.F., Parsons P.J. (2005). A critical review of biomarkers used for monitoring human exposure to lead: Advantages, limitations, and future needs. Environ. Health Perspect..

[171] Gulson B.L., Mizon K.J., Korsch M.J., Horwarth D., Phillips A., Hall J. (1996). Impact on blood lead in children and adults following relocation from their source of exposure and contribution of skeletal tissue to blood lead. Bull. Environ. Contam. Toxicol..

[172] Gulson B.L., Mahaffey K.R., Mizon K.F., Korsch M.J., Cameron M.A., Vimpani G. (1995). Contribution of tissue lead to bone lead in adult female subjects based on stable lead-isotope methods. J. Lab. Clin. Med..

[173] Gwiazda R., Campbell C., Smith D. (2005). A noninvasive isotopic approach to estimate the bone lead contribution to blood in children: Implications for assessing the efficacy of lead abatement. Environ. Health Perspect..

[174] Manton W.I., Angle C.R., Stanek K.L., Reese Y.R., Kuehnemann T.J. (2000). Acquisition and retention of lead by young children. Environ. Res..

[175] Roberts J.R., Reigart. J.R., Ebeling. M., Hulsey T.C. (2001). Time required for blood lead levels to decline in nonchelated children. Clin. Toxicol..

[176] Landrigan P.J., Todd A.C. (1994). Direct measurement of lead in bone-a promising biomarker. JAMA.

[177] O’Flaherty E.J. (1995). Physiologically based models for bone-seeking elements V: Lead absorption and disposition in childhood. Toxicol. Appl. Pharmacol..

[178] Hu H., Rabinowitz M., Smith. D. (1998). Bone lead as a biological marker in epidemiologic studies of chronic toxicity: Conceptual paradigms. Environ. Health Perspect..

[179] Nilsson U., Attewell R., Christoffersson J.O., Schütz A., Ahlgren L., Skerfving S., Mattsson S. (1991). Kinetics of lead in bone and blood after end of occupational exposure. Pharmacol. Toxicol..

[180] Price J., Grudzinski A.W., Craswell P.W., Thomas B.J. (1992). Repeated bone lead levels in Queensland, Australia—Previously a high lead environment. Arch. Environ. Health.

[181] Brito J.A., McNeill F.E., Stronach I., Webber C.E., Wells S., Richard N., Chettle D.R. (2001). Longitudinal changes in bone lead concentration: Implications for modelling of human bone lead metabolism. J. Environ. Monit..

[182] Wilker E., Korrick S., Nie L.H., Sparrow D., Vokonas P., Coull B., Wright R.O., Schwartz. J., Hu H. (2011). Longitudinal changes in bone lead levels: The VA normative aging study. J. Occup. Environ. Med..

[183] Gerhardsson L., Akantis A., Lundström N.G., Nordberg G.F., Schütz A., Skerfving S. (2005). Lead concentrations in cortical and trabecular bones in deceased smelter workers. J. Trace Elem. Med. Biol..

[184] Gerhardsson L., Englyst V., Lundström N.G., Nordberg G., Sandberg S., Steinvall F. (1995). Lead in tissues of deceased lead smelter workers. J. Trace Elem. Med. Biol..

[185] Hernandez-Avila M., Smith D., Meneses F., Sanin L.H., Hu H. (1998). The influence of bone and blood lead on plasma lead levels in environmentally exposed adults. Environ. Health Perspect..

[186] Hu H., Shih R., Rothenberg S., Schwartz B.S. (2007). The epidemiology of lead toxicity in adults: Measuring dose and consideration of other methodologic issues. Environ. Health Perspect..

[187] Shih R.A., Hu H., Weisskopf M.G., Schwartz B.S. (2007). Cumulative lead dose and cognitive function in adults: A review of studies that measured both blood lead and bone lead. Environ. Health Perspect..

[188] Farooqui Z., Bakulski K.M., Power M.C., Weisskopf M.G., Sparrow D., Spiro A., Vokonas P.S., Nie L.H., Hu H., Park S.K. (2017). Associations of cumulative Pb exposure and longitudinal changes in mini-mental status exam scores, global cognition and domains of cognition: The VA normative aging study. Environ. Res..

[189] Wright R.O., Tsaih S.W., Schwartz J., Spiro A., McDonald K., Weiss S.T., Hu H. (2003). Lead exposure biomarkers and mini-mental status exam scores in older men. Epidemiology.

[190] Zheutlin A.R., Hu H., Weisskopf M.G., Sparrow D., Vokonas P.S., Park S.K. (2018). Low-level cumulative lead and resistant hypertension: A prospective study of men participating in the Veterans Affairs normative aging study. J. Am. Heart Assoc..

[191] Hirata M., Yoshida T., Miyajima K., Kosaka H., Tabuchi T. (1995). Correlation between lead in plasma and other indicators of lead exposure among lead-exposed workers. Int. Arch. Occup. Environ. Health.

[192] Schütz A., Olsson M., Jensen A., Gerhardsson L., Börjesson J., Mattsson S., Skerfving S. (2005). Lead in finger bone, whole blood, plasma and urine in lead-smelter workers: Extended exposure range. Int. Arch. Occup. Environ. Health.

[193] Fukui Y., Miki M., Ukai H., Okamoto S., Takada S., Higashikawa K., Ikeda M. (1999). Urinary lead as a possible surrogate of blood lead among workers occupationally exposed to lead. Int. Arch. Occup. Environ. Health.

[194] Bai Y., Laenen A., Haufroid V., Nawrot T.S., Nemery B. (2019). Urinary lead in relation to combustion-derived air pollution in urban environments. A longitudinal study of an international panel. Environ. Int..

[195] Wang X., Jin P., Zhou Q., Liu S., Wang F., Xi S. (2019). Metal biomonitoring and comparative assessment in urine of workers in lead-zinc and steel-iron mining and smelting. Biol. Trace Elem. Res..

[196] Li S., Wang J., Zhang B., Liu Y., Lu T., Shi Y., Shan G., Dong L. (2018). Urinary lead concentration is an independent predictor of cancer mortality in the U.S. general population. Front. Oncol..

[197] Dudley R.E., Gammal L.M., Klaassen C.D. (1985). Cadmium-induced hepatic and renal injury in chronically exposed rats: Likely role of hepatic cadmium-metallothionein in nephrotoxicity. Toxicol. Appl. Pharmacol..

[198] Chan H.M., Zhu L.F., Zhong R., Grant D., Goyer R.A., Cherian M.G. (1993). Nephrotoxicity in rats following liver transplantation from cadmium-exposed rats. Toxicol. Appl. Pharmacol..

[199] Shaikh Z.A., Vu T.T., Zaman K. (1999). Oxidative stress as a mechanism of chronic cadmium-induced hepatoxicity and renal toxicity and protection by antioxidants. Toxicol. Appl. Pharmacol..

[200] Goyer R.A., Miller C.R., Zhu S.-Y., Victery W. (1989). Non-metallothionein-bound cadmium in the pathogenesis of cadmium nephrotoxicity in the rat. Toxicol. Appl. Pharmacol..

[201] Liu Y., Liu J., Habeebu S.S.M., Klaasen C.D. (1999). Metallothionein protects against the nephrotoxicity produced by chronic CdMT exposure. Toxicol. Sci..

[202] Vestergaard P., Shaikh Z.A. (1994). The nephrotoxicity of intravenously administered cadmium-metallothionein: Effect of dose, mode of administration, and preexisting renal cadmium burden. Toxicol. Appl. Pharmacol..

[203] Min K.S., Onosaka S., Tanaka K. (1996). Renal accumulation of cadmium and nephropathy following long-term administration of cadmium-metallothionein. Toxicol. Appl. Pharmacol..

[204] Price R.G. (1992). The role of NAG (N-acetyl-β-D-glucosaminidase) in the diagnosis of kidney disease including the monitoring of nephrotoxicity. Clin. Nephrol..

[205] Bernard A., Thielemans N., Roels H., Lauwerys R. (1995). Association between NAG-B and cadmium in urine with no evidence of a threshold. Occup. Environ. Med..

[206] Jin T., Nordberg G., Wu X., Kong Q., Wang Z., Zhuang F., Cai S. (1999). Urinary N-acetyl-beta-D-glucosaminidase isoenzymes as biomarker of renal dysfunction caused by cadmium in a general population. Environ. Res..

[207] Tassi C., Abbritti G., Mancuso F., Morucci P., Feligioni L., Muzi G. (2000). Activity and isoenzyme profile of N-acetyl-beta-D-glucosaminidase in urine from workers exposed to cadmium. Clin. Chim. Acta.

[208] Prozialeck W.C., Edwards J.R. (2010). Early biomarkers of cadmium exposure and nephrotoxicity. Biometals.

[209] Prozialeck W.C., Vaidya V.S., Liu J., Waalkes M.P., Edwards J.R., Lamar P.C., Bernard A.M., Dumont X., Bonventre J.V. (2007). Kidney injury molecule-1 is an early biomarker of cadmium nephrotoxicity. Kidney Int..

[210] Prozialeck W.C., Edwards J.R., Vaidya V.S., Bonventre J.V. (2009). Preclinical evaluation of novel urinary biomarkers of cadmium nephrotoxicity. Toxicol. Appl. Pharmacol..

[211] Prozialeck W.C., Edwards J.R., Lamar P.C., Liu J., Vaidya V.S., Bonventre J.V. (2009). Expression of kidney injury molecule-1 (Kim-1) in relation to necrosis and apoptosis during the early stages of Cd-induced proximal tubular injury. Toxicol. Appl. Pharmacol..

[212] Pennemans V., De Winter L.M., Munters E., Nawrot T.S., Van Kerhove E., Rigo J.-M., Reynders C., Dewitte H., Carleer R., Penders J. (2011). The association between urinary kidney injury molecule 1 and urinary cadmium in elderly during long-term, low-dose cadmium exposure: A pilot study. Environ. Health.

[213] Ruangyuttikarn W., Panyamoon A., Nambunmee K., Honda R., Swaddiwudhipong W., Nishijo M. (2013). Use of the kidney injury molecule-1 as a biomarker for early detection of renal tubular dysfunction in a population chronically exposed to cadmium in the environment. Springerplus.

[214] Zhang Y., Wang P., Liang X., Chuen S., Tan J., Wang J., Huang Q., Huang R., Li Z., Chen W. (2015). Associations between urinary excretion of cadmium and renal biomarkers in nonsmoking females: A cross-sectional study in rural areas of South China. Int. J. Environ. Res. Public Health.

[215] Chaumont A., Voisin C., Deumer G., Haufroid V., Annesi-Maesano I., Roels H., Thijs L., Staessen J., Bernard A. (2013). Associations of urinary cadmium with age and urinary proteins: Further evidence of physiological variations unrelated to metal accumulation and toxicity. Environ. Health Perspect..

[216] Nomiyama K., Foulkes E.C. (1977). Reabsorption of filtered cadmium-metallothionein in the rabbit kidney. Proc. Soc. Exp. Biol. Med..

[217] Tanimoto A., Hamada T., Koide O. (1993). Cell death and regeneration of renal proximal tubular cells in rats with subchronic cadmium intoxication. Toxicol. Pathol..

[218] Roels H.A., Lauwerys R.R., Buchyet J.-P., Bernard A., Chettle D.R., Harvey T.C., Al-Haddad I.K. (1981). In vivo measurement of liver and kidney cadmium in workers exposed to this metal: Its significance with respect to cadmium in blood and urine. Environ. Res..

[219] Weaver V.M., Kim N.-S., Jaar B.G., Schwartz B.S., Parsons P.J., Steuerwald A.J., Todd A.C., Simon D., Lee B.-K. (2011). Associations of low-level urine cadmium with kidney function in lead workers. Occup. Environ. Med..

[220] Buser M.C., Ingber S.Z., Raines N., Fowler D.A., Scinicariello F. (2016). Urinary and blood cadmium and lead and kidney function: NHANES 2007–2012. Int. J. Hyg. Environ. Health.

[221] Jin R., Zhu X., Shrubsole M.J., Yu C., Xia Z., Dai Q. (2018). Associations of renal function with urinary excretion of metals: Evidence from NHANES 2003–2012. Environ. Int..

[222] Kawada T., Koyama H., Suzuki S. (1989). Cadmium, NAG activity, and β2-microglobulin in the urine of cadmium pigment workers. Br. J. Ind. Med..

[223] Kawada T., Shinmyo R.R., Suzuki S. (1992). Urinary cadmium and N-acetyl-β-D-glucosaminidase excretion of inhabitants living in a cadmium-polluted area. Int. Arch. Occup. Environ. Health.

[224] Koyama H., Satoh H., Suzuki S., Tohyama C. (1992). Increased cadmium excretion and its relationship to urinary N-acetyl-β-D-glucosaminidase activity in smokers. Arch. Toxicol..

[225] Thomas L.D., Hodgson S., Nieuwenhuijsen M., Jarup L. (2009). Early kidney damage in a population exposed to cadmium and other heavy metals. Environ. Health Perspect..

[226] Wang D., Sun H., Wu Y., Zhou Z., Ding Z., Chen X., Xu Y. (2016). Tubular and glomerular kidney effects in the Chinese general population with low environmental cadmium exposure. Chemosphere.

[227] Akesson A., Lundh T., Vahter M., Bjellerup P., Lidfeldt J., Nerbrand C., Samsioe G., Strömberg U., Skerfving S. (2005). Tubular and glomerular kidney effects in Swedish women with low environmental cadmium exposure. Environ. Health Perspect..

[228] Eom S.-Y., Seo M.-N., Lee Y.-S., Park K.-S., Hong Y.-S., Sohn S.-J., Kim Y.-D., Choi B.-S., Lim J.-A., Kwon H.-J. (2017). Low-level environmental cadmium exposure induces kidney tubule damage in the general population of Korean adults. Arch. Environ. Contam. Toxicol..

[229] Swaddiwudhipong W., Limpatanachote P., Mahasakpan P., Krintratun S., Punta B., Funkhiew T. (2012). Progress in cadmium-related health effects in persons with high environmental exposure in northwestern Thailand: A five-year follow-up. Environ. Res..

[230] Piscator M. (1984). Long-term observations on tubular and glomerular function in cadmium-exposed persons. Environ. Health Perspect..

[231] Roels H.A., Lauwerys R.R., Buchyet J.P., Bernard A.M., Vos A., Oversteyns M. (1989). Health significance of cadmium induced renal dysfunction: A five year follow up. Br. J. Ind. Med..

[232] Jarup L., Persson B., Elinder C.G. (1995). Decreased glomerular filtration rate in solderers exposed to cadmium. Occup. Environ. Med..

[233] Mason H.J., Davison A.G., Wright A.L., Guthrie C.J.G., Fayers P.M., Venables K.M., Smith N.J., Chettle D.R., Franklin D.M., Scott M.C. (1988). Relations between liver cadmium, cumulative exposure, and renal function in cadmium alloy workers. Br. J. Ind. Med..

[234] Satarug S., Moore M.R. (2004). Adverse health effects of chronic exposure to low-level cadmium in foodstuffs and cigarette smoke. Environ. Health Perspect..

[235] Schnaper H.W. (2017). The tubulointerstitial pathophysiology of progressive kidney disease. Adv. Chronic Kidney Dis..

[236] Schardijn G.H.C., Statius van Eps L.W. (1987). β_2_-microglobulin: Its significance in the evaluation of renal function. Kidney Int..

[237] Hall P.W., Chung-Park M., Vacca C.V., London M., Crowley A.Q. (1982). The renal handling of beta_2_-microglobulin in the dog. Kidney Int..

[238] Gauthier C., Nguyen-Simonnet H., Vincent C., Revillard J.-P., Pellet M.V. (1984). Renal tubular absorption of β2-microglobulin. Kidney Int..

[239] Norden A.G.W., Lapsley M., Lee P.J., Pusey C.D., Scheinman S.J., Tam F.W.K., Thakker R.V., Unwin R.J., Wrong O. (2001). Glomerular protein sieving and implications for renal failure in Fanconi syndrome. Kidney Int..

[240] Wibell L., Evrin P.-E., Berggard J. (1973). Serum β_2_-microglobulin in renal disease. Nephron.

[241] Wibell L.B. (1976). Studies on β2-microglobulin in patients and normal subjects. Acta Clin. Belg..

[242] Wibell L. (1978). The serum level and urinary excretion of β2-microglobulin in health and renal disease. Pathol. Biol. (Paris).

[243] Hall P.W., Ricanati E.S. (1981). Renal handling of β2-microglobulin in renal disorders with special reference to hepatorenal syndrome. Nephron.

[244] Portman R.J., Kissane J.M., Robson A.M. (1986). Use of β2-microglobulin to diagnose tubulo-interstitial renal lesions in children. Kidney Int..

[245] Bernard A. (2004). Renal dysfunction induced by cadmium: Biomarkers of critical effects. Biometals.

[246] Nogawa K., Kobayashi E., Honda R. (1979). A study of the relationship between cadmium concentrations in urine and renal effects of cadmium. Environ. Health Perspect..

[247] Ikeda M., Ezaki T., Moriguchi J., Fukui Y., Ukai H., Okamoto S., Sakurai H. (2005). The threshold cadmium level that causes a substantial increase in β2-microglobulin in urine of general populations. Tohoku J. Exp. Med..

[248] Peterson P.A., Evrin P.-E., Berggard I. (1969). Differentiation of glomerular, tubular, and normal proteinuria: Determinations of urinary excretion of β2-microglobulin, albumin, and total protein. J. Clin. Investig..

[249] Elinder C.G., Edling C., Lindberg E., Agedal B.K., Vesterberg A. (1985). Assessment of renal function in workers previously exposed to cadmium. Br. J. Ind. Med..

[250] Norden A.G.W., Lapsley M., Unwin R.J. (2014). Urine retinol-binding protein 4: A functional biomarker of the proximal renal tubule. Adv. Clin. Chem..

[251] Blaner W.S. (1989). Retinol-binding protein: The serum transport protein for vitamin A. Endocri. Rev..

[252] Pallet N., Chauvet S., Chasse J.-F., Vincent M., Avilloach P., Levi C., Meas-Yedid V., Olivo-Marin J.-C., Nga-Matsogo D., Beaune P. (2014). Urinary retinol binding protein is a marker of the extent of interstitial kidney fibrosis. PLoS ONE.

[253] Bernard A., Vyskocyl A., Mahieu P., Lauwerys R. (1988). Effect of renal insufficiency on the concentration of free retinol-binding protein in urine and serum. Clin. Chim. Acta.

[254] Jarup L., Akesson A. (2009). Current status of cadmium as an environmental health problem. Toxicol. Appl. Pharmacol..

[255] Heymsfield S.B., Arteaga C., McManus C., Smith J., Moffitt S. (1983). Measurement of muscle mass in humans: Validity of the 24-hour urinary creatinine method. Am. J. Clin. Nutr..

[256] Jenny-Burri J., Haldimann M., Bruschweiler B.J., Bochuyd M., Burnier M., Paccaud F., Dudler V. (2015). Cadmium body burden of the Swiss population. Food Addit. Contam. Part A Chem. Anal. Control. Expo. Risk Assess..

[257] Chevalier R.L., Forbes M.S. (2008). Generation and evolution of atubular glomeruli in the progression of renal disorders. J. Am. Soc. Nephrol..

[258] Ferenbach D.A., Bonventre J.V. (2016). Acute kidney injury and chronic kidney disease: From the laboratory to the clinic. Nephrol. Ther..

[259] Zammouri A., Barbouch S., Najjar M., Aoudia R., Jaziri F., Kaaroud H., Hedri H., Abderrahim E., Goucha R., Hamida F.B. (2019). Tubulointerstitial nephritis due to sarcoidosis: Clinical, laboratory, and histological features and outcome in a cohort of 24 patients. Saudi J. Kidney Dis. Transpl..

[260] Goules A., Geetha D., Arend L.J., Baer A.N. (2019). Renal involvement in primary Sjogren’s syndrome: Natural history and treatment outcome. Clin. Exp. Rheumatol..

[261] Jasiek M., Karras A., Le Guern V., Krastinova E., Mesbah R., Faguer S., Jourde-Chiche N., Fauchais A.-L., Chiche L., Dernis E. (2017). A multicentre study of 95 biopsy-proven cases of renal disease in primary Sjogren’s syndrome. Rheumatology.

[262] Kelly C.J., Neilson E.G., Taal M.W., Chertow G.M., Marsden P.A., Skorecki K., Alan S.L., Brenner B.M. (2011). Tubulointerstitial diseases. Brenner & Rector’s The Kidney.

[263] Nath K.A. (1992). Tubulointerstitial changes as a major determinant in the progression of renal damage. Am. J. Kidney Dis..

[264] Risdon R.A., Sloper J.C., De Wardener H.E. (1968). Relationship between renal function and histological changes found in renal-biopsy specimens from patients with persistent glomerular nephritis. Lancet.

[265] Schainuck L.I., Striker G.E., Cutler R.E., Benditt E.P. (1970). Structural-functional correlations in renal disease: Part II: The correlations. Human Pathol..

[266] Bohle A., von Gise H., Mackensen-Haen S., Stark-Jakob B. (1981). The obliteration of the postglomerular capillaries and its influence upon the function of both glomeruli and tubuli. Functional interpretation of morphologic findings. Klin. Wochenschr..

[267] Baba H., Tsuneyama K., Kumada T., Aoshima K., Imura J. (2014). Histopathological analysis for osteomalacia and tubulopathy in itai-itai disease. J. Toxicol. Sci..

[268] Yasuda M., Miwa A., Kitagawa M. (1995). Morphometric studies of renal lesions in itai-itai disease: Chronic cadmium nephropathy. Nephron.

[269] Saito H., Shioji R., Hurukawa Y., Nagai K., Arikawa T. (1977). Cadmium-induced proximal tubular dysfunction in a cadmium-polluted area. Contrib. Nephrol..

[270] Nogawa K., Ishizaki A., Fukushima M., Shibata I., Hagino N. (1975). Studies on the women with acquired Fanconi syndrome observed in the Ichi River basin polluted by cadmium. Environ. Res..

[271] Rappaport S.M., Smith M.T. (2010). Environment and disease risks. Science.

[272] Lee J., Oh S., Kang H., Kim S., Lee G., Li L., Kim C.T., An J.N., Oh Y.K., Lim C.S. (2020). Environment-wide association study of CKD. Clin. J. Am. Soc. Nephrol..

[273] Soderland P., Lovekar S., Weiner D.E., Brooks D.R., Kaufman J.S. (2010). Chronic kidney disease associated with environmental toxins and exposures. Adv. Chronic Kidney Dis..

[274] Chevalier R.L. (2016). The proximal tubule is the primary target of injury and progression of kidney disease: Role of the glomerulotubular junction. Am. J. Physiol. Renal. Physiol..

[275] Crowley S.D., Coffman T.M. (2014). The inextricable role of the kidney in hypertension. J. Clin. Investig..

[276] Nakhoul N., Batuman V. (2011). Role of proximal tubules in the pathogenesis of kidney disease. Contrib. Nephrol..

[277] De Nicola L., Zoccali C. (2016). Chronic kidney disease prevalence in the general population: Heterogeneity and concerns. Nephrol. Dial. Transplant..

[278] Glassock R.J., David G., Warnock D.G., Delanaye P. (2017). The global burden of chronic kidney disease: Estimates, variability and pitfalls. Nat. Rev. Nephrol..

[279] Chen T.K., Knicely D.H., Grams M.E. (2019). Chronic kidney disease diagnosis and management: A review. JAMA.

[280] Lees J.S., Welsh C.E., Celis-Morales C.A., Mackay D., Lewsey J., Gray S.R., Lyall D.M., Cleland J.G., Gill J.M.R., Jhund P.S. (2019). Glomerular filtration rate by differing measures, albuminuria and prediction of cardiovascular disease, mortality and end-stage kidney disease. Nat. Med..

[281] Sarnak M.J., Amann K., Bangalore S., Cavalcante J.L., Charytan D.M., Craig J.C., Gill J.S., Hlatky M.A., Jardine A.G., Landmesser U. (2019). Chronic kidney disease and coronary artery disease: JACC state-of-the-art review. J. Am. Coll. Cardiol..

[282] Liu Y., Lv P., Jin H., Cui W., Niu C., Zhao M., Fan C., Teng Y., Pan B., Peng Q. (2016). Association between low estimated glomerular filtration rate and risk of cerebral small-vessel diseases: A meta-analysis. J. Stroke Cerebrovasc. Dis..

[283] Kelly D.M., Rothwell P.M. (2019). Does chronic kidney disease predict stroke risk independent of blood pressure? A systematic review and meta-regression. Stroke.

[284] Akoudad S., Sedaghat S., Hofman A., Koudstaal P.J., van der Lugt A., Ikram M.A., Vernooij M.W. (2015). Kidney function and cerebral small vessel disease in the general population. Int. J. Stroke.

[285] Sedaghat S., Ding J., Eiriksdottir G., van Buchem M.A., Sigurdsson S., Ikram M.A., Meirelles O., Gudnason V., Levey A.S., Launer L.J. (2018). The AGES-Reykjavik study suggests that change in kidney measures is associated with subclinical brain pathology in older community-dwelling persons. Kidney Int..

[286] White C.A., Allen C.M., Akbari A., Collier C.P., Holland D.C., Day A.G., Knoll G.A. (2019). Comparison of the new and traditional CKD-EPI GFR estimation equations with urinary inulin clearance: A study of equation performance. Clin. Chim. Acta.

[287] George C., Mogueo A., Okpechi I., Echouffo-Tcheugui J.B., Kengne A.P. (2017). Chronic kidney disease in low-income to middle-income countries: The case for increased screening. BMJ Glob. Health.

[288] Payton M., Hu H., Sparrow D., Weiss S.T. (1994). Low-level lead exposure and renal function in the Normative Aging Study. Am. J. Epidemiol..

[289] Navas-Acien A., Tellez-Plaza M., Guallar E., Muntner P., Silbergeld E., Jaar B., Weaver V. (2009). Blood cadmium and lead and chronic kidney disease in US adults: A joint analysis. Am. J. Epidemiol..

[290] Zhu X.J., Wang J.J., Mao J.H., Shu Q., Du L.Z. (2019). Relationships between cadmium, lead and mercury levels and albuminuria: Results from the National Health and Nutrition Examination Survey Database 2009−2012. Am. J. Epidemiol..

[291] Harari F., Sallsten G., Christensson A., Petkovic M., Hedblad B., Forsgard N., Melander O., Nilsson P.M., Borné Y., Engström G. (2018). Blood lead levels and decreased kidney function in a population-based cohort. Am. J. Kidney Dis..

[292] Sommar J.N., Svensson M.K., Björ B.M., Elmståhl S.I., Hallmans G., Lundh T., Schön S.M., Skerfving S., Bergdahl I.A. (2013). End-stage renal disease and low level exposure to lead, cadmium and mercury; a population-based, prospective nested case-referent study in Sweden. Environ. Health.

[293] Satarug S., Swaddiwudhipong W., Ruangyuttikarn W., Nishijo M., Ruiz P. (2013). Modeling cadmium exposures in low- and high-exposure areas in Thailand. Environ. Health Perspect..

[294] Swaddiwudhipong W., Nguntra P., Kaewnate Y., Mahasakpan P., Limpatanachote P., Aunjai T., Jeekeeree W., Punta B., Funkhiew T., Phopueng I. (2015). Human health effects from cadmium exposure: Comparison between persons living in cadmium-contaminated and non-contaminated areas in northwestern Thailand. Southeast Asian J. Trop. Med. Public Health.

[295] Sun Y., Sun D., Zhou Z., Zhu G., Lei L., Zhang H., Chang X., Jin T. (2008). Estimation of benchmark dose for bone damage and renal dysfunction in a Chinese male population occupationally exposed to lead. Ann. Occup. Hyg..

[296] Chen X., Zhu G., Wang Z., Zhou H., He P., Liu Y., Jin T. (2019). The association between lead and cadmium co-exposure and renal dysfunction. Ecotoxicol. Environ. Saf..

[297] Kim N.H., Hyun Y.Y., Lee K.B., Chang Y., Ryu S., Oh K.H., Ahn C. (2015). Environmental heavy metal exposure and chronic kidney disease in the general population. J. Korean Med. Sci..

[298] Myong J.P., Kim H.R., Baker D., Choi B. (2012). Blood cadmium and moderate-to-severe glomerular dysfunction in Korean adults: Analysis of KNHANES 2005−2008 data. Int. Arch. Occup. Environ. Health.

[299] Chung S., Chung J.H., Kim S.J., Koh E.S., Yoon H.E., Park C.W., Chang Y.S., Shin S.J. (2014). Blood lead and cadmium levels and renal function in Korean adults. Clin. Exp. Nephrol..

[300] Lim H., Lim J.A., Choi J.H., Kwon H.J., Ha M., Kim H., Park J.D. (2016). Associations of low environmental exposure to multiple metals with renal tubular impairment in Korean adults. Toxicol. Res..

[301] Hambach R., Lison D., D’Haese P.C., Weyler J., De Graef E., De Schryver A., Lamberts L.V., van Sprundel M. (2013). Co-exposure to lead increases the renal response to low levels of cadmium in metallurgy workers. Toxicol. Lett..

[302] Ferraro P.M., Sturniolo A., Naticchia A., D’Alonzo S., Gambaro G. (2012). Temporal trend of cadmium exposure in the United States population suggests gender specificities. Intern. Med. J..

[303] Agarwal S., Zaman T., Tuzcu E.M., Kapadia S.R. (2011). Heavy metals and cardiovascular disease: Results from the National Health and Nutrition Examination Survey (NHANES) 1999–2006. Angiology.

[304] Hecht E.M., Arheart K.L., Lee D.J., Hennekens C.H., Hlaing W.M. (2016). Interrelation of cadmium, smoking, and cardiovascular disease (from the National Health and Nutrition Examination Survey). Am. J. Cardiol..

[305] Hecht E.M., Arheart K.L., Lee D.J., Hennekens C.H., Hlaing W.M. (2018). Interrelationships of cadmium, smoking, and angina in the National Health and Nutrition Examination Survey, a cross-sectional study. Cardiology.

[306] Chen C., Xun P., Tsinovoi C., McClure L.A., Brockman J., MacDonald L., Cushman M., Cai J., Kamendulis L., Mackey J. (2018). Urinary cadmium concentration and the risk of ischemic stroke. Neurology.

[307] Gallagher C.M., Chen J.J., Kovach J.S. (2010). Environmental cadmium and breast cancer risk. Aging (Albany NY).

[308] Tellez-Plaza M., Navas-Acien A., Menke A., Crainiceanu C.M., Pastor-Barriuso R., Guallar E. (2012). Cadmium exposure and all-cause and cardiovascular mortality in the U.S. general population. Environ. Health Perspect..

[309] Menke A., Muntner P., Silbergeld E.K., Platz E.A., Guallar E. (2009). Cadmium levels in urine and mortality among U.S. adults. Environ. Health Perspect..

[310] Adams S.V., Passarelli M.N., Newcomb P.A. (2012). Cadmium exposure and cancer mortality in the Third National Health and Nutrition Examination Survey cohort. Occup. Environ. Med..

[311] Hyder O., Chung M., Cosgrove D., Herman J.M., Li Z., Firoozmand A., Gurakar A., Koteish A., Pawlik T.M. (2013). Cadmium exposure and liver disease among US adults. J. Gastrointest. Surg..

[312] Min J.Y., Min K.B. (2016). Blood cadmium levels and Alzheimer’s disease mortality risk in older US adults. Environ. Health.

[313] Peng Q., Bakulski K.M., Nan B., Park S.K. (2017). Cadmium and Alzheimer’s disease mortality in U.S. adults: Updated evidence with a urinary biomarker and extended follow-up time. Environ. Res..

[314] Moberg L., Nilsson P.M., Samsioe G., Sallsten G., Barregard L., Engström G., Borgfeldt C. (2017). Increased blood cadmium levels were not associated with increased fracture risk but with increased total mortality in women: The Malmö Diet and Cancer Study. Osteoporos Int..

[315] Deering K.E., Callan A.C., Prince R.L., Lim W.H., Thompson P.L., Lewis J.R., Hinwood A.L., Devine A. (2018). Low-level cadmium exposure and cardiovascular outcomes in elderly Australian women: A cohort study. Int. J. Hyg. Environ. Health.

[316] Suwazono Y., Nogawa K., Morikawa Y., Nishijo M., Kobayashi E., Kido T., Nakagawa H., Nogawa K. (2015). All-cause mortality increased by environmental cadmium exposure in the Japanese general population in cadmium non-polluted areas. J. Appl. Toxicol..

[317] Watanabe Y., Nogawa K., Nishijo M., Sakurai M., Ishizaki M., Morikawa Y., Kido T., Nakagawa H., Suwazono Y. (2020). Relationship between cancer mortality and environmental cadmium exposure in the general Japanese population in cadmium non-polluted areas. Int. J. Hyg. Environ. Health.

[318] Maruzeni S., Nishijo M., Nakamura K., Morikawa Y., Sakurai M., Nakashima M., Kido T., Okamoto R., Nogawa K., Suwazono Y. (2014). Mortality and causes of deaths of inhabitants with renal dysfunction induced by cadmium exposure of the polluted Jinzu River basin, Toyama, Japan; a 26-year follow-up. Environ. Health.

[319] Nishijo M., Nakagawa H., Suwazono Y., Nogawa K., Sakurai M., Ishizaki M., Kido T. (2018). Cancer mortality in residents of the cadmium-polluted Jinzu River basin in Toyama, Japan. Toxics.

[320] van Bemmel D.M., Li Y., McLean J., Chang M.H., Dowling N.F., Graubard B., Rajaraman P. (2011). Blood lead levels, ALAD gene polymorphisms, and mortality. Epidemiology.

[321] Lanphear B.P., Rauch S., Auinger P., Allen R.W., Hornung R.W. (2018). Low-level lead exposure and mortality in US adults: A population-based cohort study. Lancet Public Health.

[322] Aoki Y., Brody D.J., Flegal K.M. (2016). Blood lead and other metal biomarkers as risk factors for cardiovascular disease mortality. Medicine.

[323] Kim M.G., Ryoo J.H., Chang S.J., Kim C.B., Park J.K., Koh S.B., Ahn Y.S. (2015). Blood lead levels and cause-specific mortality of inorganic lead-exposed workers in South Korea. PLoS ONE.

[324] Min Y.S., Ahn Y.S. (2017). The association between blood lead levels and cardiovascular diseases among lead-exposed male workers. Scand. J. Work Environ. Health.

